# Stem Growth and Dehydration Responses of Mediterranean Tree Species to Atmospheric and Soil Drought

**DOI:** 10.1111/pce.15177

**Published:** 2024-10-03

**Authors:** Roberto L. Salomón, J. Julio Camarero

**Affiliations:** ^1^ Departamento de Sistemas y Recursos Naturales, Research Group FORESCENT Universidad Politécnica de Madrid Madrid Spain; ^2^ Instituto Pirenaico de Ecología (IPE‐CSIC) Zaragoza Spain

**Keywords:** bimodality, drought, high‐resolution dendrometers, stem desiccation, stem swelling‐shrinkage, tree water deficit, xylem

## Abstract

Stem growth responses to soil and atmospheric drought are critical to forecasting the tree carbon sink strength. Yet, responses of drought‐prone forests remain uncertain despite global aridification trends. Stem diameter variations at an hourly resolution were monitored in five Mediterranean tree species from a mesic and a xeric site for 6 and 12 years. Stem growth and dehydration responses to soil (REW) and atmospheric (VPD) drought were explored at different timescales. Annually, growth was determined by the number of growing days and hours. Seasonally, growth was bimodal (autumn growth ≈ 8%–18% of annual growth), varying among species and sites across the hydrometeorological space, while dehydration consistently responded to REW. Sub‐daily, substantial growth occurred during daytime, with nighttime‐to‐daytime ratios ranging between 1.2 and 3.5 (*Arbutus unedo* ≈ *Quercus faginea* < *Quercus ilex* < *Pinus halepensis* in the mesic site, and *Juniperus thurifera* < *P. halepensis* in the xeric site). Overall, time windows favourable for growth were limited by soil (rather than atmospheric) drought, modulating annual and seasonal growth in Mediterranean species, and stems maintained non‐negligible growth during daytime. These patterns contrast with observations from wetter or cooler biomes, demonstrating the growth plasticity of drought‐prone species to more arid climate conditions.

## Summary statement

Yearly, seasonal and sub‐daily stem growth patterns of Mediterranean tree species contrast with observations from wetter biomes, denoting a high growth plasticity of drought‐prone species to a changing climate.

## 1 Introduction

Forests worldwide annually sequester about one‐third of anthropogenic CO_2_ emissions (Ruehr et al. 2023), with tree stem wood constituting the largest living carbon sink of these terrestrial ecosystems (Poorter et al. 2012). As tree stem growth depends on temperature, vapour pressure deficit (VPD) and water availability (Babst et al. 2019), understanding tree growth responses to these environmental drivers at variable time scales (yearly, seasonal and sub‐daily) is needed to forecast forest carbon budgets and their feedback to climate change. Inter‐annual variability of tree growth is commonly assessed from tree ring analyses (Fritts 1976). Nevertheless, yearly data lack the temporal resolution required to gain a mechanistic understanding of tree growth processes at the seasonal and sub‐daily time scales (Steppe, Sterck, and Deslauriers 2015). Automatic dendrometers can capture stem diameter variations (Δ*D*) at a high spatial (< 1 µm) and temporal (sub‐hourly) resolution, which makes them an outstanding tool for the study of tree ecophysiological responses to short‐term climatic variations (De Swaef et al. 2015; Steppe, Sterck, and Deslauriers 2015; Zweifel 2016).

However, translating dendrometer readings to stem growth rates is not straightforward. Stem Δ*D* integrates multiple signals challenging to disentangle: irreversible plastic growth, elastic changes in the stem water pools and elastic osmotically‐driven fluctuations in the phloem (Mencuccini et al. 2013). The zero‐growth concept (Zweifel et al. 2016) has become the preferential approach to separate these signals due to its biological robustness in explaining stem growth physiology and its straightforward applicability, which allows for near real‐time assessment of growth rates (GRO) and circumvents more complex modelling procedures requiring ancillary data (Steppe et al. 2006; Mencuccini et al. 2013; Chan et al. 2016; Salomón et al. 2020). The zero‐growth concept assumes that trees cannot grow during dry periods of stem shrinkage (Zweifel et al. 2016). Under this theoretical framework, a second metric known as the tree water deficit (TWD) can be calculated as the difference between the instantaneous stem diameter and its preceding maximum. Therefore, the TWD informs about the depletion of stem water pools in terms of the stem linear elastic shrinkage. As stem water pools match the water atmospheric demand with the root supply, functioning as a hydraulic capacitor (Meinzer et al. 2009; Salomón et al. 2017), TWD has proven to be a reliable proxy of stem water status (Dietrich, Zweifel, and Kahmen 2018; Drew et al. 2011).

Dendrometer affordability, improvements in its applicability and automatisation of data processing routines (Knüsel et al. 2021) have led to remarkable progress in understanding stem Δ*D* in recent years. Likewise, efforts in compiling long‐term data sets have proven helpful in evaluating the interspecific heterogeneity of stem Δ*D* at large spatial scales (e.g., Zweifel, Etzold, et al. 2021; Salomón, Peters, et al. 2022). However, our understanding of stem Δ*D* is biased mainly towards the physiological behaviour of temperate species. Illustratively, Zhou et al. (2023) compiled dendrometer data to evaluate the seasonal effect of temperature, precipitation, VPD and soil moisture on stem growth. Of the 130 species × site combinations reviewed in this study, 94, 18, 10 and 8 were classified as temperate, tropical, boreal and subtropical. Overall, comprehensive Δ*D* data sets have shown that, on an annual time scale, growth is explained by intermittent temporal windows favourable for growth rather than the total length of the growing season (Etzold et al. 2022). At the seasonal time scale, growth is commonly unimodal, at least in boreal, temperate and subtropical biomes, mainly concentrated at the beginning of the growing season when environmental conditions are optimal for growth, depending on species‐specific or biome‐specific requirements (Etzold et al. 2022; Zhou et al. 2023). However, bimodal patterns, with growth peaks in spring and autumn, have also been detected in Mediterranean trees and shrubs (Camarero, Olano, and Parras 2010; Camarero, Rubio‐Cuadrado, and Gazol 2021; Valeriano et al. 2023; Albrecht et al. 2023). At the sub‐daily time scale, growth has been consistently observed to occur at night, when VPD reductions allow xylem tension relaxation and the subsequent build‐up of turgor pressure in cambial cells (Zweifel, Sterck, et al. 2021; Zhou et al. 2023).

Water availability is the dominant limiting factor (over temperature and solar radiation) for tree production worldwide (Churkina and Running 1998), and the drought‐driven limitation to growth is expected to increase with global warming (Babst et al. 2019). This aridification trend places drought‐prone Mediterranean tree species in a hotspot, and therefore, their growth response to more frequent and severe drought events becomes critical in predicting global forest productivity and climatic feedback (Cherubini et al. 2003). Yet, highly resolved Δ*D* variations and their underlying drivers in Mediterranean tree species remain comparatively unexplored. Observational studies have commonly focused on one (e.g., Vieira et al. 2013, 2022; Lempereur et al. 2015) or few (e.g., Sánchez‐Costa, Poyatos, and Sabaté 2015; Aldea et al. 2018; Güney et al. 2020; Camarero 2022; Albrecht et al. 2023) co‐occurring species, commonly from the same site, with (sub)hourly dendrometer time series rarely exceeding 3 years. Growth and dehydration patterns observed in boreal, temperate and tropical tree species may differ from those of drought‐prone Mediterranean regions, where soil water availability is more limiting for growth, and consequently, growth patterns might be more plastic and multimodal. Therefore, there is a need to verify whether stem Δ*D* in temperate (Zweifel, Sterck, et al. 2021; Etzold et al. 2022) and subtropical (Zhou et al. 2023) species at different time scales hold across Mediterranean species and are governed by similar environmental cues, which would facilitate the implementation of universal physiological mechanisms in large‐scale models of forest productivity.

To fill this knowledge gap, long‐term (6–12 years) and highly resolved dendrometer data series of five Mediterranean tree species located in two sites of contrasted water availability were analysed to test the following hypotheses: (i) At the inter‐annual time scale, stem growth is proportional to the duration (not the intensity) of time‐windows favourable for growth and thus inversely related to the duration of the drought‐induced shrinkage period. (ii) At the seasonal time scale, a bimodal growth pattern is driven by soil water availability before summer drought and after autumnal rains. (iii) At the sub‐daily timescale, nighttime relaxation of the atmospheric evaporative demand allows turgor‐mediated stem growth. We discuss these three hypotheses within the context of species‐specific water use regulation and functional behaviour.

## 2 Materials and Methods

### 2.1 Study Sites

We selected two sites with contrasting climatic conditions located in Aragón, north‐eastern Spain: a mesic site (Agüero, 42.31° N, 0.80° W, 748 m a.s.l.) and a xeric site (Peñaflor, 41.78° N, 0.72° W, 363 m a.s.l.). The mean annual temperature and total annual precipitation are 13.0°C and 775 mm and 14.8°C and 358 mm in the mesic and xeric sites, respectively. The period with negative water balance goes from July to September and May to October in the mesic and xeric sites, respectively. The mean soil moisture content, measured at 10–15 cm using a time domain reflectometry probe (ThetaProbe Soil Moisture Sensor, Delta‐T, Cambridge, UK) from 2007 to 2011, was 20.1% and 10.8% in the mesic and xeric sites, respectively (see more details in Camarero, Rubio‐Cuadrado, and Gazol 2021). Soil moisture content was highest during late winter to early spring (February to May; 30%–35%) and lowest from summer to early autumn (July to September; 5%–10%). Figure S1 in Supporting Information shows the study sites' location, aerial views and climate diagrams.

The mesic site is a woodland dominated by oaks (the evergreen *Quercus ilex* L. subsp. *ballota* (Desf.) Samp. and the winter‐deciduous *Quercus faginea* Lam.), the evergreen *Arbutus unedo* L. and shade‐intolerant conifers including pines (mainly *Pinus halepensis* Mill. and *Pinus nigra* J.F. Arn.) and junipers (*Juniperus oxycedrus* L.). Most oak trees are multi‐stemmed due to their historical use as coppices. The soil in this area is a Calcisol developed on Miocene clays, and the bedrock is formed by calcareous sandstone. The xeric site consists of an open shrubland with scattered pine (*P. halepensis*) and juniper (*Juniperus thurifera* L.) stands. The dominant shrubs are *Juniperus phoenicea* L., *Pistacia lentiscus* L., *Rhamnus alaternus* L. and *Quercus coccifera* L. (Camarero, Olano, and Parras 2010). Soils are basic, with gypsum and marl forming the parent rock material. No local anthropogenic disturbance (wildfire, thinning or logging) has affected the study sites at least since the 1970s.

### 2.2 Study Species

We selected mature trees of dominant species growing in the mesic (*Q. ilex*, *Q. faginea*, *A. unedo* and *P. halepensis*) and xeric (*P. halepensis* and *J. thurifera*) sites. The five study species present different wood types and leaf habits (Table 1). The evergreen conifers *P. halepensis* and *J. thurifera* are found across the western Mediterranean Basin under xeric conditions, often in sites with poor and rocky soils, and display a high drought tolerance (Camarero, Olano, and Parras 2010). *A. unedo* is an evergreen tree or shrub forming diffuse‐porous wood and widely distributed throughout the Mediterranean Basin in mild sites. The two oaks are found in the western Mediterranean Basin, with *Q. ilex* and *Q. faginea* forming diffuse‐porous and ring‐porous wood and occupying drier and moister sites, respectively (Montserrat‐Martí et al. 2009). According to these authors, *Q. faginea* shows an earlier leaf flushing than *Q. ilex*, while *Q. ilex* tends to show a longer autumn growth period, particularly in coastal mild sites. The study species show a prominent growth peak in spring, but some of them are facultative bimodal (e.g., *P. halepensis*, *J. thurifera* and *Q. ilex*), showing a minor growth peak in autumn (Camarero, Olano, and Parras 2010, Camarero, Rubio‐Cuadrado, and Gazol 2021).

**Table 1:** Sites, species, ecological features and functional traits of monitored trees.

Site	Species	Leaf habit	Wood type	Specific leaf area (mm^2^ mg^−1^)	Wood density (mg cm^−3^)	Dbh (cm)	No. individuals	Height (m)	Age at 1.3 m (years)
Mesic site	*Arbutus unedo*	Evergreen	Diffuse‐porous	6.55	0.63	9.1 ± 0.3	2	5.0 ± 0.1	41 ± 2
	*Pinus halepensis*	Evergreen	Conifer	3.85	0.53	18.0 ± 1.1	2	6.8 ± 0.5	45 ± 2
	*Quercus faginea*	Deciduous	Ring‐porous	7.40	0.70	12.8 ± 0.6	2	7.2 ± 0.5	39 ± 3
	*Quercus ilex*	Evergreen	Diffuse‐porous	4.19	0.71	10.6 ± 0.5	2	5.4 ± 0.4	36 ± 3
Xeric site	*Juniperus thurifera*	Evergreen	Conifer	3.14	0.62	12.4 ± 0.6	5	5.2 ± 0.3	45 ± 3
	*P. halepensis*	Evergreen	Conifer	3.70	0.58	16.2 ± 0.8	6	6.6 ± 0.4	67 ± 5

*Note:* Values are means ± standard error. Dbh is the diameter at breast height (measured at 1.3 m). Data on functional traits were obtained from Granda, Gazol, and Camarero (2018).

### 2.3 Climate Data

In each site, automated weather stations (Campbell CR‐1000, Campbell Sci., Logan, USA) were installed to measure, among others, air temperature (T_AIR_, °C), relative humidity (RH_AIR_, %), volumetric soil water content measured at 10–15 cm (SWC, %) and rainfall (L m^−2^) every 15 min. Climatic data were aggregated into hourly values (see Alday et al. 2020). The atmospheric VPD was calculated from T_AIR_ and RH_AIR_ readings as a metric of the atmospheric drought (Allen et al. 1998). The range of SWC largely differed between the mesic (12%–61%) and the xeric (3%–22%) sites. Therefore, the relative extractable water (REW, Equation 1) was calculated as a metric of soil drought to facilitate comparison between sites (Granier et al. 2007). The REW was calculated by scaling the SWC to the site‐specific field capacity (SWC_MAX_) and the lowest soil moisture (SWC_MIN_) recorded during the monitored period:

(1) $$\text{REW}\;=\;\left({\text{SWC}\;–\;\text{SWC}}_{\mathrm{MIN}}\right){/(\text{SWC}}_{\mathrm{MAX}}{–\;\text{SWC}}_{\mathrm{MIN}}).$$

Hourly resolved climatic data for the mesic and xeric sites were available for the 2008–2020 and 2017–2019 years, respectively (Supporting Information: Figures S2 and S3).

### 2.4 Stem Diameter Variations

Changes in tree stem circumference were monitored using automatic band dendrometers (DRL26C, EMS Brno, Czech Republic) (see details in Camarero 2022) in two individuals per species in the mesic site and 5–6 individuals per species in the xeric site (Table 1). Dendrometers were placed at 1.3 m and recorded variations in stem circumference every 30 min with 1 μm resolution. These values were converted into hourly diameter variations (Δ*D*) to match climatic data resolution, assuming a circular stem shape. Before installing dendrometers, we carefully removed the dead bark and measured the diameter at breast height (dbh, measured at 1.3 m) and the height of the stems using tapes and laser rangefinders, respectively. Dendrometers were installed in September 2008, and measurements obtained in the first 4 months after installation were discarded. Dendrometer data were taken for the 2009–2014 years in the mesic site and for the 2009–2013 and 2016–2022 periods in the xeric site (Supporting Information: Figures S4 and S5). In early 2023, cores were taken at 1.3 m using a Pressler increment borer to estimate tree age (Table 1).

### 2.5 Data Analyses

R software (version 4.2.2) was used for data analyses. Dendrometer data was processed with the *treenetproc* package (Haeni et al. 2020). In cases of temporal gaps and constant (artefactual) values exceeding 1 week during the growing season, the whole annual time series was excluded from analyses, as these could bias annual estimates of growth and TWD. Growth (GRO) and TWD at different time scales (yearly, seasonal and sub‐daily) were calculated from the cleaned dendrometer time series according to the zero‐growth concept (Zweifel et al. 2016). Therefore, instantaneous GRO was estimated as the difference between the stem diameter and its preceding value, being zero during shrinkage periods (when this difference was negative).

**Figure 1:**
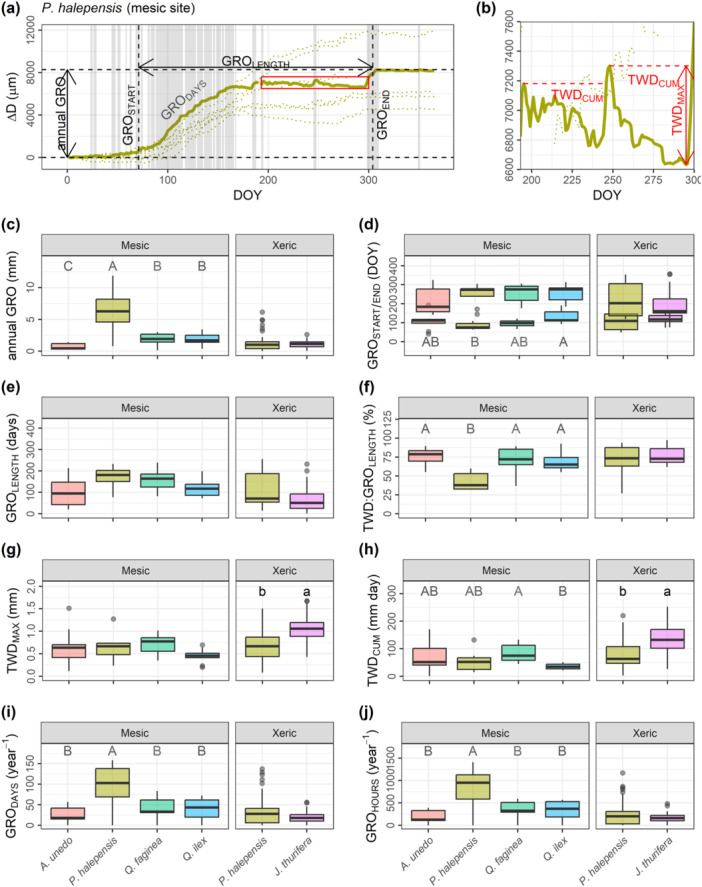
Example of highly‐resolved dendrometer data obtained from a *Pinus halepensis* tree located in the mesic site (a, b), and boxplots of yearly metrics per tree species and site (c–j). See the data analyses section for the abbreviation and the calculation of yearly metrics. Centerlines, box limits and whiskers represent the median, upper and lower quartiles and extremes, excluding outliers (beyond the 1.5 × interquartile range), thus reflecting inter‐annual variability within measurement sets. Different capital and lowercase letters denote inter‐specific differences in the mesic and xeric sites, respectively.

At the yearly timescale, several metrics were derived from the dendrometer and the climatic time series: cumulative annual growth (annual GRO), start and end of the growing season (GRO_START_ and GRO_END_; day of the year—DOY—for which 5% and 95% of the annual GRO is completed), length of the growing season (GRO_LENGTH_ = GRO_END_ − GRO_START_), number of growing days (GRO_DAYS_) and growing hours (GRO_HOURS_), maximum tree water deficit (TWD_MAX_), cumulative tree water deficit (TWD_CUM_) and the duration of the TWD period within the growing season (TWD:GRO_LENGTH_). See Figure 1a,b for an illustrative example of some of these metrics. GRO_DAYS_ and GRO_HOURS_ were estimated by summing up the number of (daily or hourly) observations exceeding the previous one during non‐shrinkage periods (when GRO > 0). Inter‐specific differences within each site were tested via non‐parametric Wilcox tests. These dendrometer‐derived yearly metrics were regressed against annual GRO independently per species × site combination (‘measurement set’, hereafter), adjusting linear mixed models considering the tree as a random factor with the *lmer* function from the *lme4* package (see Supporting Information: Notes S1 for the detailed model code). Additional linear mixed models were adjusted by pooling measurement sets and also including yearly climatic metrics as fixed factors (Supporting Information: Notes S1). For this, cumulative REW and cumulative VPD were estimated as proxies of annual soil and atmospheric drought considering the whole year or restricted to the growing season (according to the median GRO_LENGTH_ per measurement set). Marginal and conditional *R* ^2^ values, accounting for the variance of fixed and fixed plus random factors, were calculated using the package *performanc*e (Nakagawa and Schielzeth 2013).

At the seasonal timescale, daily GRO and TWD were estimated considering the sub‐daily maxima (99th percentile to exclude outliers) per day. To quantify the contribution of bimodal peaks to annual growth, the 14‐day running average was calculated to smooth GRO and TWD daily time series (Etzold et al. 2022) and the spring‐to‐autumn growth transition was set the DOY when the maximum TWD was reached. Seasonal variability in GRO and TWD was assessed across the hydrometeorological space (HMS hereafter) defined by soil drought (REW) and atmospheric drought (VPD) proxies (Zweifel, Sterck, et al. 2021; Salomón, Peters, et al. 2022) by normalising GRO and TWD daily values to the tree‐specific maxima (99th percentile) registered throughout the time series (all years included). The seasonal HMS was defined by the median (50th percentile) sub‐daily values of VPD and REW per day and site, also considering the whole time series. First, to quantitatively assess the relative weight of REW, VPD and their additive and interactive effect on daily normalised GRO and TWD, linear mixed models were adjusted considering tree and year as crossed random factors (Notes S1), and the *partR2* package was used for *R* ^2^ partitioning (Stoffel, Nakagawa, and Schielzeth 2021). Note that the *partR2* package requires *lme4* models, precluding the specification of autocorrelation structures. Secondly, local polynomial regression (*loess* function) was adjusted against daily VPD and REW as predictors of tree‐specific daily normalised GRO and TWD (Zweifel, Sterck, et al. 2021), applying a smoothing parameter *α* = 0.75 (default value) and quadratic (two‐degree) polynomials (Notes S1). For this, site‐specific HMS was estimated with the *chull* function, excluding the 1% of points further from the convex hull centre to discard from analyses extreme hydrometeorological conditions such as heavy rain events leading to soil water saturation and rapid drain.

At the sub‐daily time scale, the hourly growth rate (hourly GRO), probability and contribution to annual GRO were calculated per measurement set considering the whole time series. The hourly GRO was estimated considering growing hours (GRO > 0) according to the zero growth concept (Zweifel et al. 2016). The hourly growth probability (%) was estimated as the number of growing hours relative to the total number of hours during the monitored period. The hourly contribution to the annual growth was calculated as the cumulative growth occurring at a given hour (at any DOY) relative to the annual GRO. Growth probability and contribution were first estimated per tree × year time series, and then, the median trend per measurement set was estimated. Nighttime‐to‐daytime ratios were estimated by averaging nighttime (from 21:00 to 7:00 h) and daytime (from 8:00 to 20:00 h) hourly values. In addition, the hourly variation in stem diameter setting the zero point at midnight (24 h) was estimated throughout the study period. The sub‐daily Δ*D* pattern was separately estimated considering all days and only growing days. Sub‐daily variability in growth across the HMS was assessed using the normalised hourly GRO relative to the tree‐specific maxima (99th percentile) registered throughout the study period, including observations without growth in the analyses (hourly GRO = 0). For this, local polynomial regression was first adjusted against time (hour) and VPD, second against time and REW and third against VPD and REW as predictors of normalised hourly GRO applying the abovementioned parameters (Supporting Information: Notes S1).

## 3 Results

### 3.1 Variability in Annual Stem Growth Across Species and Sites

Stem annual GRO was commonly below 5 mm, except for *P. halepensis* in the mesic site (Figure 1c). The start of the growing season (GRO_START_) in the mesic site was the earliest for *P. halepensis* and the latest for *Q. ilex*. No differences in GRO_START_ were observed in the xeric site (Figure 1d). The end (GRO_END_) and length (GRO_LENGTH_) of the growing season did not differ among species in any site (Figure 1d,f), with median GRO_LENGTH_ ranging between 51 and 181 days for xeric *J. thurifera* and mesic *P. halepensis*, respectively. The percentage of shrinkage days within the growing season (TWD:GRO_LENGTH_) was lowest for the mesic *P. halepensis* (38%), with all other measurement sets showing median percentages above 65% (Figure 1f). TWD_MAX_ and TWD_CUM_ were higher in *J. thurifera* than in *P. halepensis* in the xeric site. In the mesic site, TWD_CUM_ was higher in *Q. faginea* than in *Q. ilex* (Figure 1g,h). GRO_DAYS_ and GRO_HOURS_ were the highest for *P. halepensis* in the mesic site (Figure 1i,j), and no differences between species were observed in the xeric site. When considering the only species monitored at both sites, *P. halepensis*, GRO‐related metrics were overall higher for the mesic pines, while TWD‐related metrics were slightly higher for the xeric ones.

**Figure 2:**
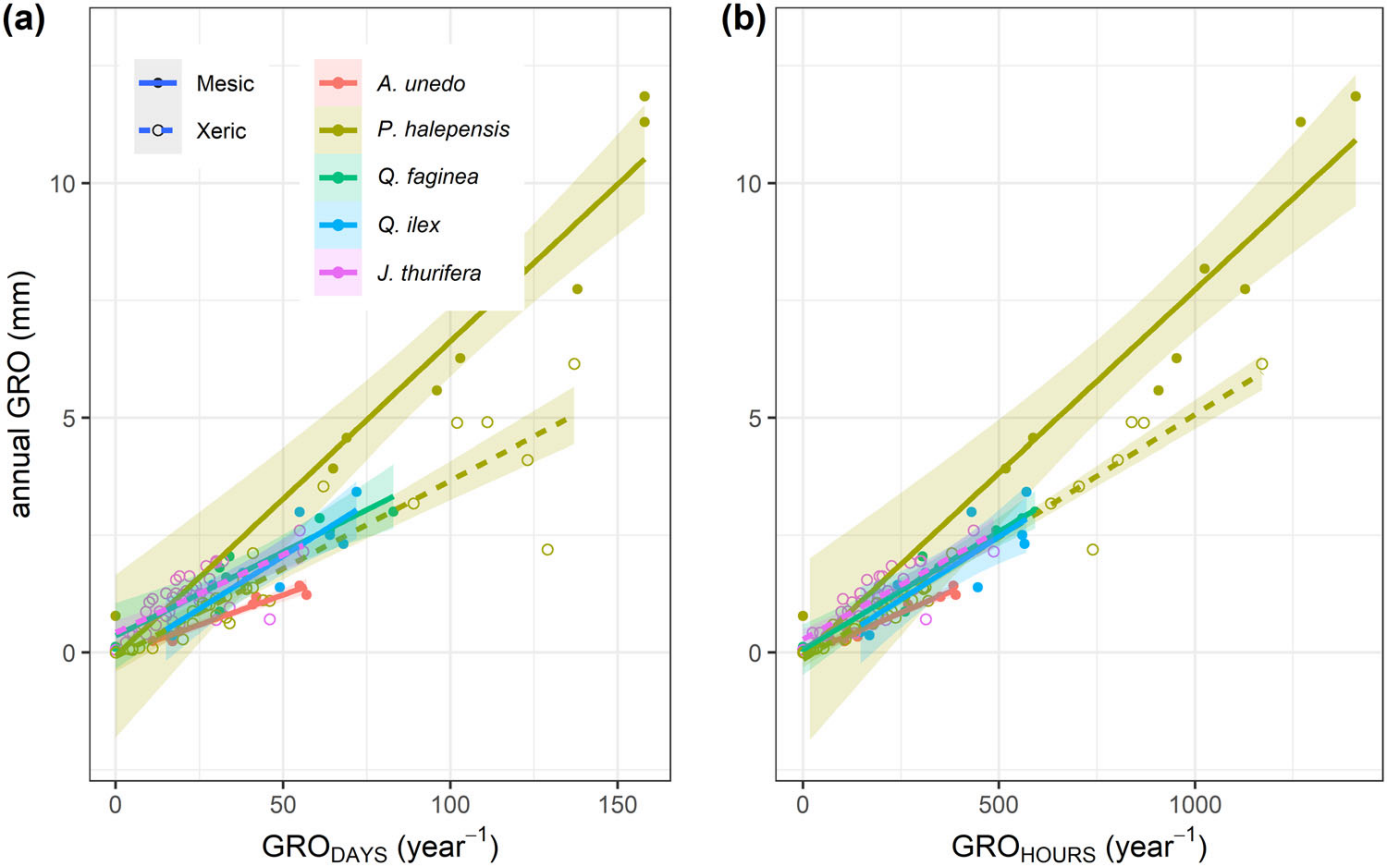
Relationship of annual growth with the number of growing days (a) and hours (b) per tree species and site. Significant (*p* < 0.05) linear relationships were observed for all measurement sets. [Color figure can be viewed at wileyonlinelibrary.com]

The best predictors of annual GRO were GRO_DAYS_ and GRO_HOURS_, for which positive and consistent relationships were observed across measurement sets (Figure 2), and their marginal *R* ^2^ were 0.75 and 0.86, respectively (Table 2). The length of the growing season (GRO_LENGTH_) and the fraction of shrinkage days within the growing season (TWD:GRO_LENGTH_) were also significantly related to annual GRO, but their marginal *R* ^2^ values were comparatively lower (0.11 and 0.19, respectively). Shrinkage‐related (TWD_MAX_ and TWD_CUM_) and phenological‐related (GRO_START_ and GRO_END_) metrics were the weakest predictors of annual GRO (marginal *R* ^2^ < 0.05). Cumulative REW and VPD did not explain annual GRO when considering whole‐year conditions (marginal *R* ^2^ < 0.01). However, when REW_CUM_ and VPD_CUM_ were restricted to the growing season, marginal *R* ^2^ increased to 0.34 and 0.08, respectively (Table 2). Accordingly, REW_CUM_ predicted GRO_DAYS_ better than VPD_CUM_ (marginal *R* ^2^ = 0.30 and 0.21).

**Table 2:** Slope, *p* values and conditional and marginal *R* ^2^ values of linear mixed models testing the relationship between annual growth and potential yearly drivers.

	GRO_START_	GRO_END_	GRO_LENGTH_	TWD_CUM_	TWD_MAX_	TWD:GRO_LENGTH_	GRO_DAYS_	GRO_HOURS_	REW_CUM_	VPD_CUM_
Slope	−0.01	0.01	0.01	0	−0.81	−0.06	0.05	0.01	0.06	0.01
*p* value	0.12	0	0	0.1	0.07	0	0	0	0	0.12
Conditional *R* ^2^	0.64	0.71	0.69	0.63	0.65	0.57	0.88	0.94	0.74	0.73
Marginal *R* ^2^	0.02	0.04	0.11	0.01	0.02	0.19	0.75	0.86	0.34	0.08

*Note:* See Section 2.4 for the abbreviation and the calculation of yearly metrics. Mixed models were adjusted pooling species and considering the tree as a random factor. Climatic metrics (REW_CUM_ and VPD_CUM_) were estimated by summing daily values restricted to the growing season.

### 3.2 Seasonal Variability in Tree Stem Growth and Dehydration

**Figure 3:**
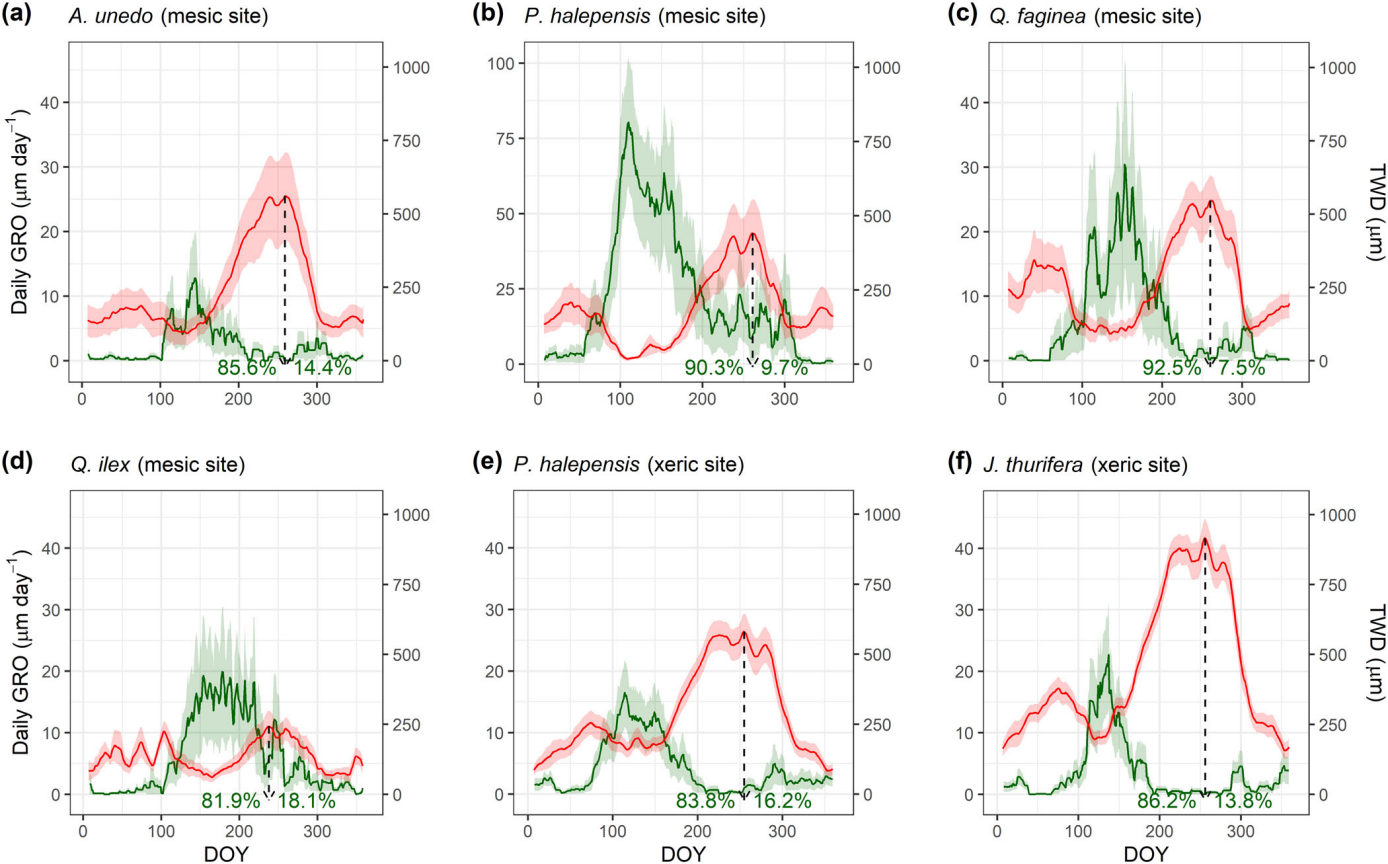
Seasonal patterns of daily growth rate (GRO, green lines) and tree water deficit (TWD, red lines) per tree species and site (a–f). Continuous lines and shaded areas show the 14‐day running average average values and standard error. The horizontal axes show the day of the year (DOY), and the vertical arrows indicate the DOY when the maximum TWD was reached. The contribution of spring and autumn peaks to the annual growth are shown as percentages next to these arrows. The *y*‐axis for *Pinus halepensis* growth in the mesic site is larger for visual clarity. [Color figure can be viewed at wileyonlinelibrary.com]

The mean daily GRO and TWD throughout the year per measurement set are shown in Figure 3. Seasonal resumption (GRO_START_) and cessation (GRO_END_) of stem growth confirm the results shown in Figure 1d. Across species, growth rates were overall highest during spring and early summer and progressively declined during summer, showing the opposite pattern to TWD. The largest TWD was reached during late summer (DOY 238–261) for all measurement sets. At this time, growth cessation was abrupt for the xeric species, while growth reductions were more gradual for the mesic species, particularly for *P. halepensis*, for which complete growth cessation did not occur. A second and relatively modest growth peak of variable intensity simultaneous with TWD relaxation was registered in autumn. The contribution of these autumnal growth peaks to annual growth varied between 7.5% and 18.1% across mesic species (Figure 3a–d) and between 13.8% and 16.2% in xeric ones (Figure 3e,f).

**Figure 4:**
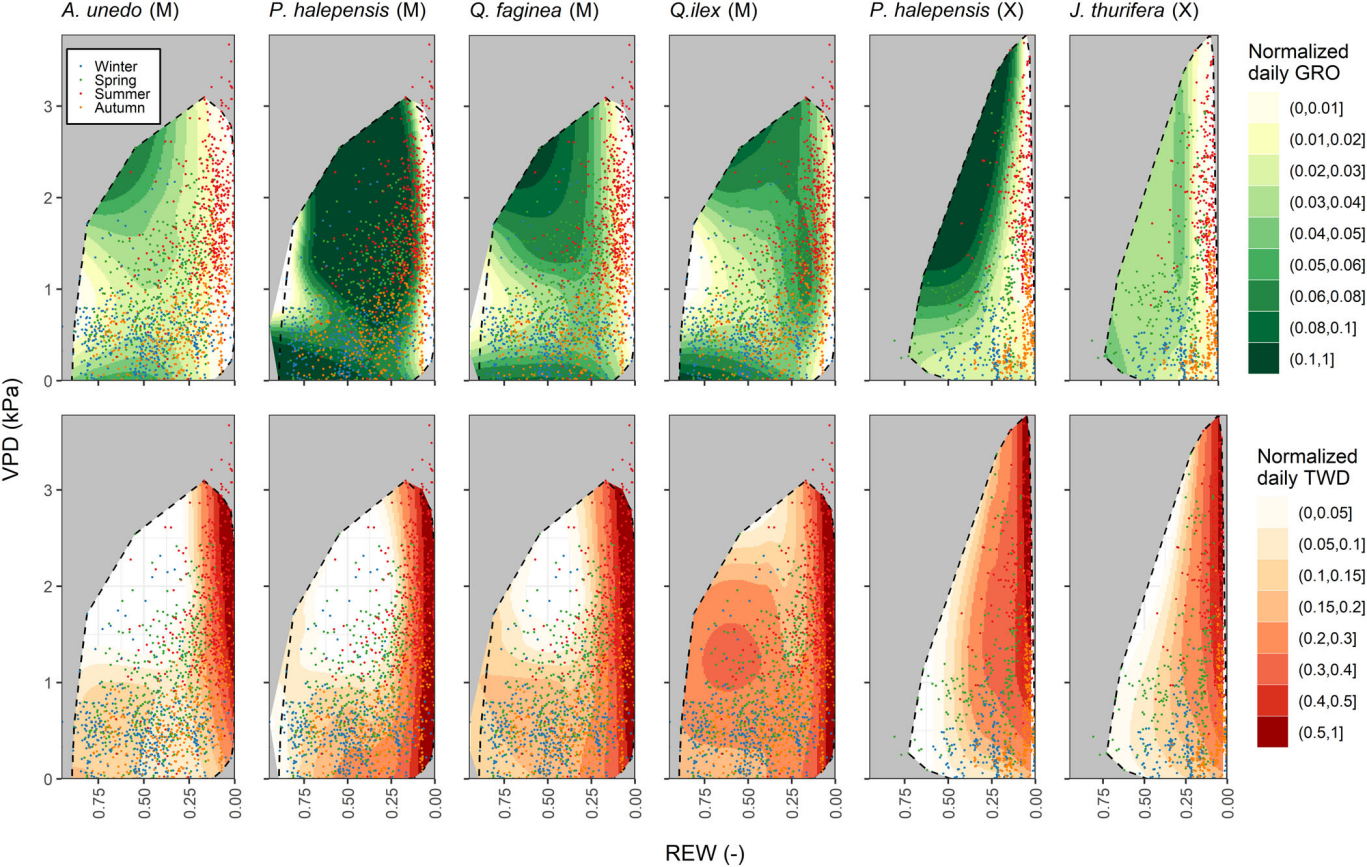
Daily normalised growth (GRO, upper panels) and tree water deficit (TWD, lower panels) per tree species and site across the hydrometeorological space (HMS), defined by atmospheric (VPD) and soil (REW) drought metrics. Normalised GRO and TWD refer to the tree‐specific maximum value (99th percentile) throughout the study period. The grey areas show the environmental conditions outside the site‐specific HMS, and the dashed polygons show the species‐specific HMS in the study site. The colour graduation differs between normalised GRO and TWD for better data visualisation. Data points are shown distinguishing among seasons. HMS regions with low point density denote relatively high model uncertainty and vice‐versa. ‘M’ stands for ‘mesic site’ and ‘X’ for ‘xeric site’. [Color figure can be viewed at wileyonlinelibrary.com]

The variance of normalised daily GRO linearly explained by REW and VPD, including their additive and interactive effects, was very low across measurement sets (marginal *R* ^2^ < 0.03; Supporting Information: Figure S6). For the normalised daily TWD, the marginal *R* ^2^ increased (from 0.07 to 0.34), with REW partial *R* ^2^ ranking first in most cases (Supporting Information: Figure S6). Seasonal variability in GRO and TWD across the HMS is shown in Figure 4 (see species‐ and site‐specific HMS in Supporting Information: Figure S7). As expected, GRO and TWD patterns were roughly opposite for all measurement sets. Overall, for the mesic species (less apparent for *P. halepensis*), relatively high normalised daily GRO occurred with (i) non‐dried soils (REW > 0.25) and high atmospheric demand (VPD > 2 kPa) and (ii) non‐dried soils but low atmospheric demand (VPD < 0.5 kPa). These two contrasting regions of the HMS occur (i) during spring and early summer and (ii) after autumn rains, illustrating the environmental requirements for bimodal growth seasonality. *P. halepensis* exceeded 10% of the maximum growth (normalised GRO > 0.1) across most of the HMS, while *A. unedo* had the most limiting hydrometeorological conditions favourable for growth. Oak species showed intermediate patterns. For the xeric species, the normalised GRO of *P. halepensis* increased bidirectionally with VPD and REW. For *J. thurifera*, normalised GRO remained stable and relatively low across most of the HMS, uniquely limited by a soil water threshold (REW ≈ 0.1). Less evident inter‐specific differences were observed for normalised daily TWD. In the mesic site, normalised TWD rapidly increased as REW dropped below 0.2. In the xeric site, the TWD increase was more gradual as soils dried out. Note, however, that the soil water available for the plant partly depends on the soil texture influencing the soil water potential; thus, inter‐site REW comparisons should be taken cautiously.

### 3.3 Sub‐Daily Variability in Tree Stem Growth and Dehydration

**Figure 5:**
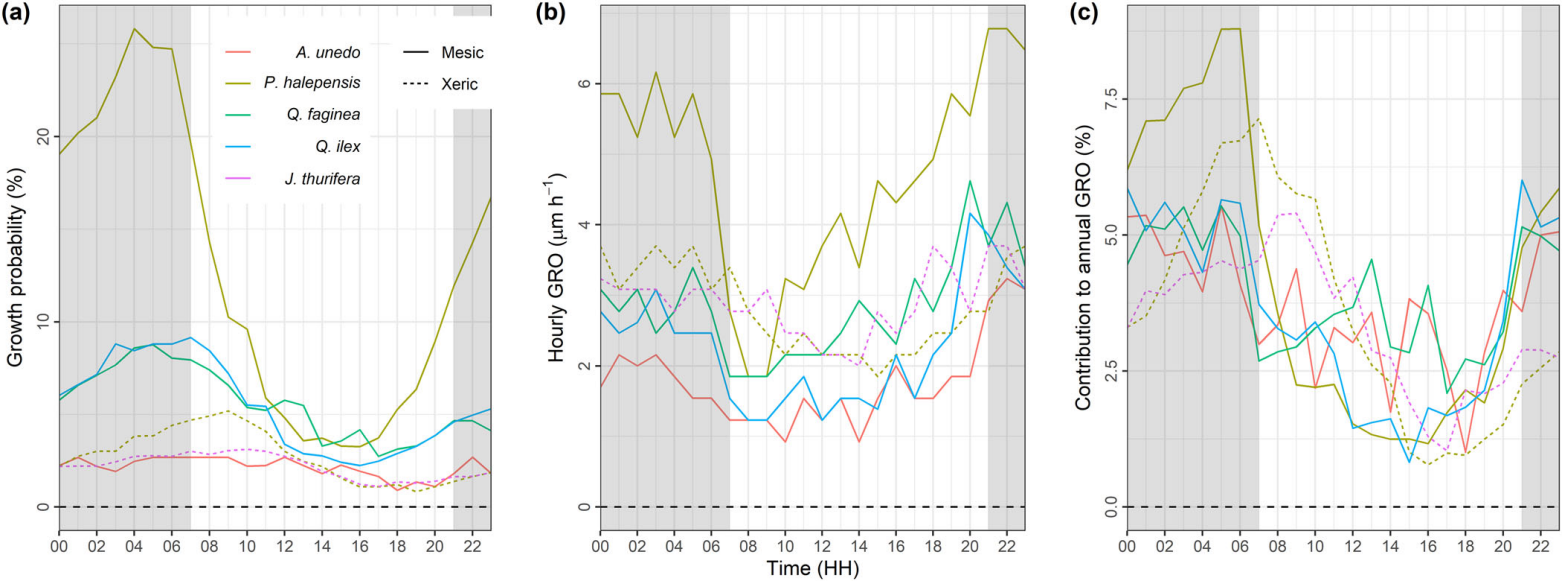
Sub‐daily patterns of growth probability (a), rate (b) and contribution to annual growth (c) per species and site. Hourly GRO (b) is calculated excluding non‐growing hours. Lines show the median of each measurement set pooling data from tree × year time series. Continuous and dashed lines show mesic and xeric sites, respectively. [Color figure can be viewed at wileyonlinelibrary.com]

The growth probability was the highest for the mesic *P. halepensis*, particularly noticeable at nighttime when the median percentage reached 20.2%. This probability decreased to 5.3% during the daytime, resulting in a 3.8‐fold reduction (Figure 5a, Table 3). The growth probability for the two oak species was around 7% at nighttime, ca. two times higher than at daytime. For *A. unedo, J. thurifera* and xeric *P. halepensis*, the average nighttime growth probability was lower (2%–3%), as it was the nighttime‐to‐daytime reduction (ratios < 1.4). The hourly GRO rate was also highest for the mesic *P. halepensis*, with median values fluctuating between 5.6 µm h^−1^ at nighttime and 3.9 µm h^−1^ at daytime. Hourly GRO for the other measurement sets was lower, with average values below 3.5 µm h^−1^. The nighttime‐to‐daytime reduction in hourly GRO rate was comparatively low, as denoted by ratios consistently below 1.5 (Figure 5b, Table 3). Consequently, the hourly contribution to annual GRO was driven mainly by sub‐daily growth probability rather than sub‐daily GRO rate. The nighttime‐to‐daytime contribution ratios were 3.5 for mesic *P. halepensis*, 2.3 for *Q. ilex*, 1.6 for xeric *P. halepensis*, 1.5 for *A. unedo* and *Q. faginea* and 1.2 for *J. thurifera* (Figure 5c, Table 3).

**Table 3:** Hourly growth probability (%), rate (µm h^−1^) and contribution to annual growth (%) averaged during nighttime (from 21:00 to 7:00 h) and daytime (from 8:00 to 20:00 h) hours. The nighttime‐to‐daytime ratio of these variables is shown.

Site	Species	Nighttime			Daytime			Night‐to‐day ratio		
		Probability	Rate	Contribution	Probability	Rate	Contribution	Probability	Rate	Contribution
Mesic	*Arbutus unedo*	2.47	2.13	4.57	2.20	1.46	3.02	1.12	1.46	1.51
	*Pinus halepensis*	20.19	5.63	6.79	5.27	3.94	1.96	3.83	1.43	3.46
	*Quercus faginea*	7.12	3.05	4.82	4.17	2.66	3.18	1.71	1.15	1.51
	*Quercus ilex*	7.16	2.75	5.22	3.27	1.85	2.23	2.19	1.48	2.34
Xeric	*P. halepensis*	3.01	3.4	4.56	2.19	2.32	2.79	1.38	1.47	1.63
	*Juniperus thurifera*	2.20	3.15	3.79	1.92	2.69	3.07	1.14	1.17	1.23

*Note:* Nighttime and daytime values are averaged from the hourly trends shown in Figure 5.

**Figure 6:**
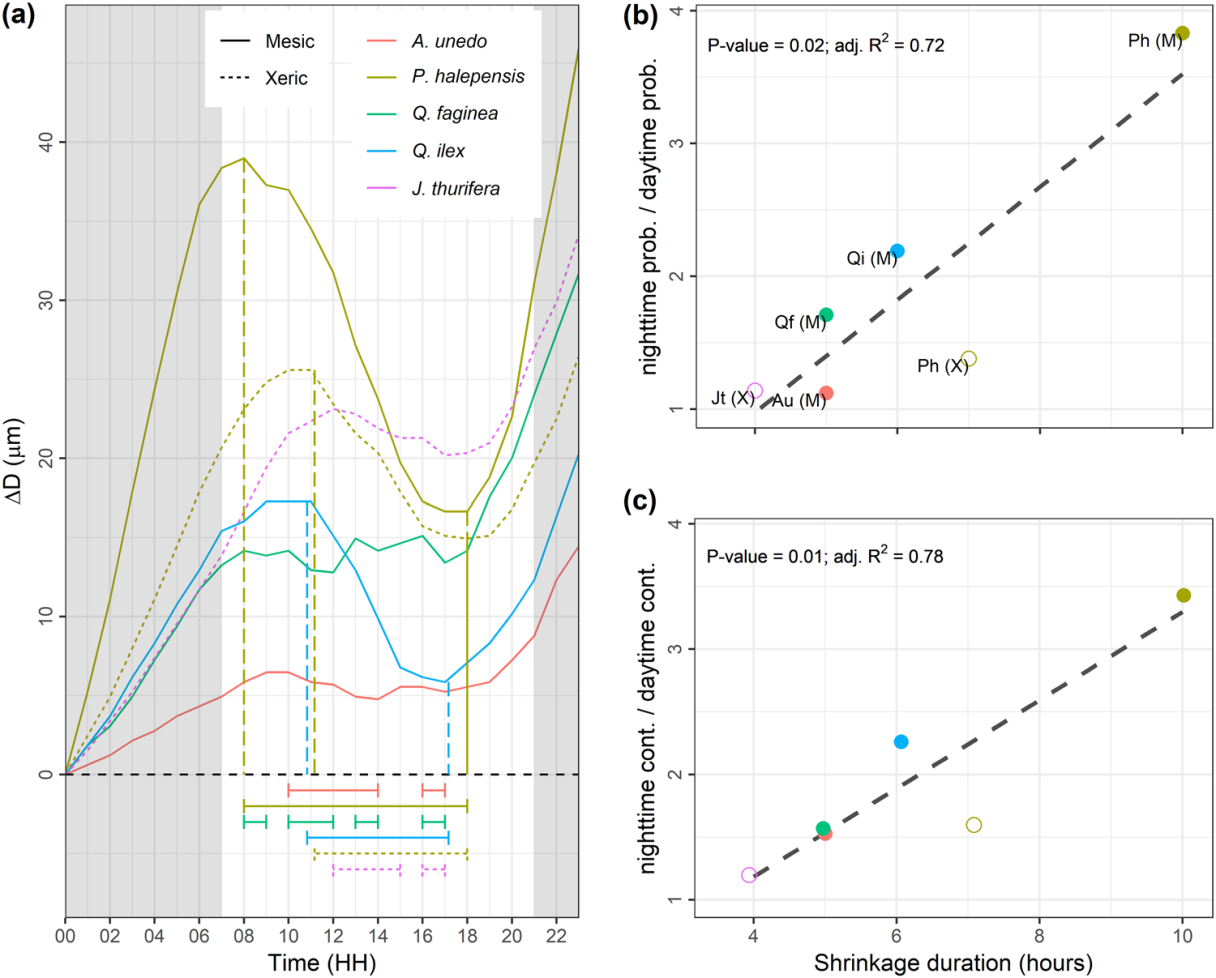
Sub‐daily patterns of stem diameter variations (Δ*D*) zeroed at midnight during growing days per species and site (a), and the relation between the number of shrinkage hours and the nighttime‐to‐daytime ratios of growth probability (b) and contribution to annual growth (c). Subdaily extremes (vertical dashed lines) delimiting the shrinkage period (horizontal segments) are shown; in cases of intermittent shrinkage, vertical lines are omitted for visual clarity (a). Linear models were fitted considering one point per measurement set (species × site combination) using data shown in Table 3. Text labels denote the species: *Arbutus unedo* (Au), *Pinus halepensis* (Ph), *Quercus faginea* (Qf), *Quercus ilex* (Qi) and *Juniperus thurifera* (Jt); in parenthesis, ‘M’ stands for ‘mesic site’ and ‘X’ for ‘xeric site’. Points are jittered along both axes in (c) to avoid overlapping between *Q. faginea* and *A. unedo*. [Color figure can be viewed at wileyonlinelibrary.com]

Regarding the sub‐daily Δ*D* zeroed at midnight, when all days were considered for analyses, the first local maximum was reached between 7:00 and 13:00 h, depending on the measurement set, and the subsequent local minimum between 19:00 and 00:00 h (Supporting Information: Figure S8). When uniquely growing days were considered, the daytime shrinkage was reduced, more markedly for *A. unedo*, *Q. faginea* and *J. thurifera,* which maintained roughly constant stem diameter with intermittent shrinkage periods (Figure 6a). Interestingly, the number of shrinkage hours was linearly related to the nighttime‐to‐daytime ratios of growth probability (Figure 6b) and contribution (Figure 6c) across measurement sets (*p* < 0.05). These nighttime‐to‐daytime ratios were not related to average intrinsic (dbh, height and age) or functional (wood density and specific leaf area) tree traits (*p* > 0.1), nor to species‐specific water potential thresholds of xylem embolism and corresponding safety margins (*p* > 0.1) measured elsewhere (data set compiled from Choat et al. 2012 and Pausas et al. 2015). However, the detection of significant relations might be hindered by the limited available measurement sets (*n* = 6) and, in the case of hydraulic traits, by the statistical noise ascribed to their intra‐specific variability.

**Figure 7:**
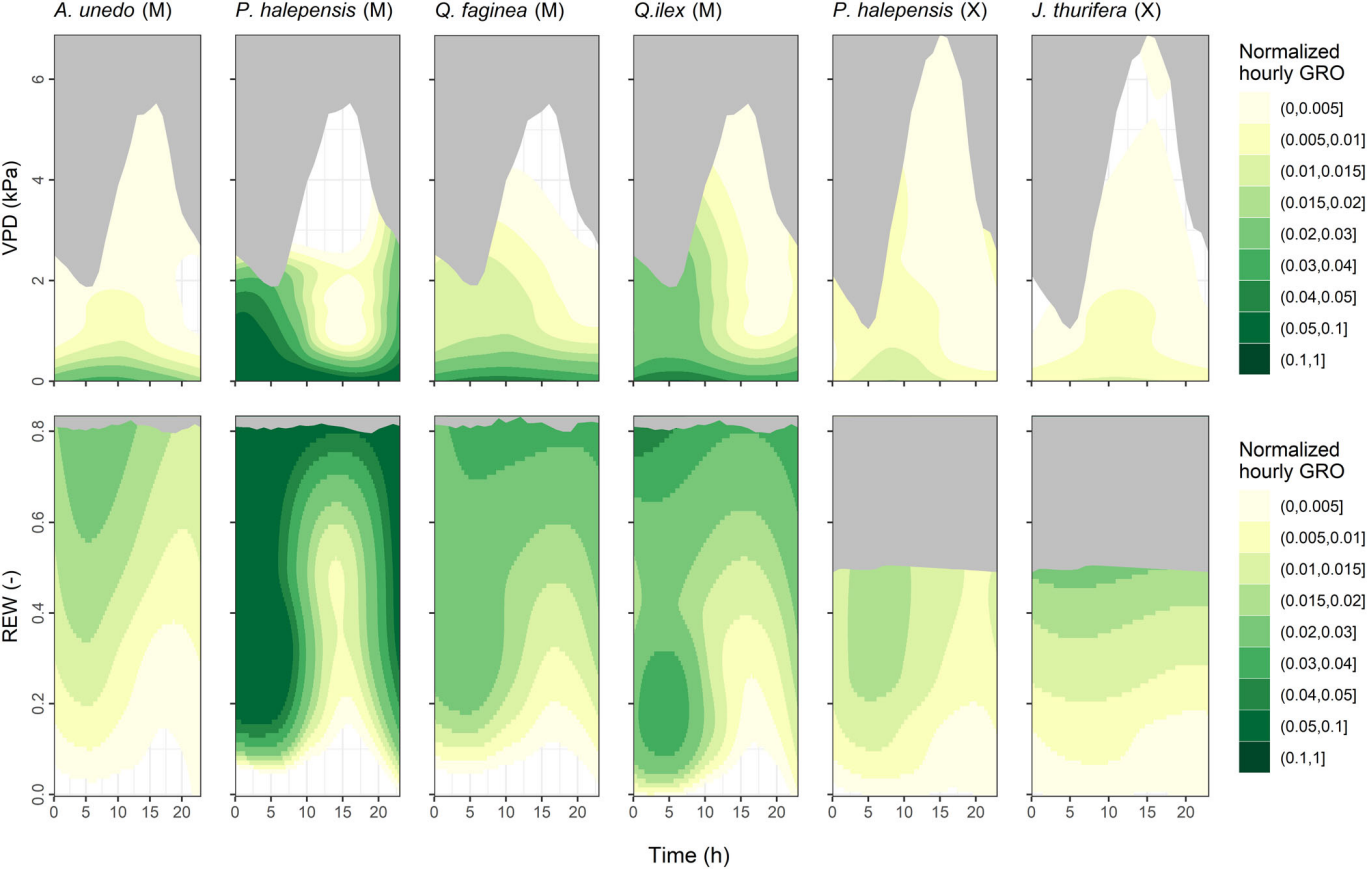
Hourly normalised growth per tree species and site as a function of time (hour) and vapour pressure deficit (VPD, upper panels) or time and relative extractable water (REW, lower panels). Normalised hourly GRO refers to the tree‐specific maximum value (99th percentile) throughout the study period. The grey areas denote the time‐environmental conditions outside the site‐specific space. ‘M’ stands for ‘mesic site’ and ‘X’ for ‘xeric site’. [Color figure can be viewed at wileyonlinelibrary.com]

The influence of VPD and REW on normalised hourly GRO differed among measurement sets (Figure 7). In the mesic site, the growth dependency on the time of the day was as follows: *P. halepensis* > *Q. ilex* > *Q. faginea* > *A. unedo*, meaning that *P. halepensis* showed the strongest circadian growth patterns. This gradient implies that hourly GRO was largely limited to nighttime for *P. halepensis* regardless of VPD and REW. By contrast, growth was more stable during the 24 h for *A. unedo*. Overall, the xeric species experienced weak circadian regulation. *P. halepensis* grew more around dawn for a given REW, while *J. thurifera* showed more stable growth regardless of the time of day. Finally, the normalised hourly GRO was predicted as a function of VPD and REW using the hourly time series (Supporting Information: Figure S9). In the mesic site, normalised GRO increased with decreasing VPD overall, a pattern more apparent for *A. unedo*. By contrast, *P. halepensis* and *Q. ilex* maintained moderate GRO at relatively high VPD under non‐completely dried‐out soils. In the xeric site, normalised GRO responded bidirectionally to VPD and REW, which was more apparent for *J. thurifera*.

## 4 Discussion

### 4.1 Yearly and Seasonal Variation in Stem Growth and Dehydration

Comparisons among species show higher annual GRO, GRO_DAYS_ and GRO_HOURS_ of mesic *P. halepensis* compared to sympatric species (Figure 1), suggesting a primary role of the duration of growth time windows in determining stem annual growth. More relevant, significant relations between GRO_DAYS_ and GRO_HOURS_ as predictive variables of annual GRO were consistently observed for all measurement sets (Figure 2). The marginal *R* ^2^ of these relations were 0.75 and 0.86, respectively, denoting that inter‐annual variability in stem yearly GRO can be mainly attributed to the number of days and hours favourable for growth, with the predictive power of these temporal metrics increasing with their resolution. As for our observations, GRO_DAYS_ largely explained the variability in annual GRO across seven temperate tree species (Etzold et al. 2022). Consistent relations across species and bioclimatic regions support the hypothesis of a ubiquitous regulation of annual GRO by the duration of the time windows favourable for growth, more important than the overall length of the growing season (Etzold et al. 2022) or the growth rate during these windows (Michelot et al. 2012).

We hypothesised that drought‐induced stem shrinkage would be a reliable predictor of annual growth reductions. However, metrics related to TWD intensity (TWD_CUM_ and TWD_MAX_) barely explained any variability in annual GRO (marginal *R* ^2^ ≤ 0.02), while TWD duration (TWD:GRO_LENGTH_) had moderate explanatory power (marginal *R* ^2^ = 0.19). This observation suggests a more relevant role of the drought timing within the growing period than the drought intensity (Gao et al. 2018; Salomón, Peters, et al. 2022). In Mediterranean climates, stem growth usually starts in spring, stops during the summer dry period and resumes after the first autumn rains (Montserrat‐Martí et al. 2009; Camarero, Rubio‐Cuadrado, and Gazol 2021; Camarero, Olano, and Parras 2010; Lempereur et al. 2015; Aldea et al. 2018; Valeriano et al. 2023; Albrecht et al. 2023). This bimodal seasonal pattern was observed here across species and sites, with autumn growth accounting for 8%−18% of the annual growth (Figure 3). This autumnal contribution might be even higher in terms of biomass, as dendrometers cannot detect cell wall deposition occurring at the end of the growing season. Phenological‐related metrics (GRO_START_ and GRO_END_) have been observed to influence annual growth in boreal (Lupi et al. 2010) and temperate regions (Michelot et al. 2012; Delpierre et al. 2016; Huang et al. 2020). However, their limited influence in the studied Mediterranean species (marginal *R* ^2^ ≤ 0.04) is unsurprising, as drought‐driven shrinkage substantially limited time windows favourable for growth during most of the growing season (TWD:GRO_LENGTH_ = 65%–78%), except for mesic *P. halepensis* trees. The complementary percentages reflecting the days of effective growth within the growing season (35%–22%) were, therefore, lower than those of temperate species (30%–80%; Etzold et al. 2022). Climatically, growth limitations were mainly imposed by soil water shortage rather than atmospheric drought, as indicated by the closer coupling of REW_CUM_ (compared to VPD_CUM_) with both annual GRO and GRO_DAYS_. This observation aligns with large‐scale analyses in which the vegetation productivity in dry sites was more sensitive to soil water limitations than to the atmospheric demand, in contrast to expectations from wetter sites (Novick et al. 2016; Kannenberg et al. 2024).

### 4.2 Climatic Drivers of the Seasonality in Stem Growth and Dehydration

At the seasonal timescale, the variability of normalised daily GRO linearly explained by hydrometeorological conditions was minor (Supporting Information: Figure S6), highlighting the complex and non‐linear growth response to environmental drivers, also dependent on unaccounted‐for factors such as the source‐sink balance and the wood phenological rhythm. By contrast, the higher variability explained in TWD predictions suggests that stem dehydration is a more biophysical process that hydrometeorological conditions (especially REW) can linearly explain to a greater extent (Figure 4 and Supporting Information: Figure S6). The limited power of linear mixed models to predict GRO denotes the need for non‐linear regression techniques for a more precise detection of site‐ and species‐specific environmental conditions favourable for growth (Figure 4). Bimodal seasonal patterns were observed across the HMS of the species in the mesic site, with two separate areas favourable for growth. All species showed the highest daily GRO in spring and early summer when VPD and REW were high before soil water depletion (i.e., upper‐left HMS). At this time, the high availability of water and photosynthates enables the build‐up of cell turgor (Lockhart 1965; Steppe et al. 2006; Fatichi et al. 2019; Peters et al. 2021) required for cell cambium division and elongation during the early phases of wood growth (Cuny et al. 2015). A second smaller growth period occurred after the first autumn rains. At this time, soil water pools were partly replenished, and despite a low atmospheric demand (i.e., lower‐left HMS) attributable to cloudy days during mild autumn months, the carbon requirements for growth were likely met by the seasonal replenishment of carbohydrate reserves. By contrast, daily GRO was impeded by excessively dried soils (REW < 0.1) occurring during summer, irrespective of the atmospheric water demand. Under these conditions, soil water depletion impeded the replenishment of stem water pools necessary to maintain cell turgor and growth (Zweifel et al. 2016), confirmed by the largest stem shrinkage registered across the HMS (lower panels in Figure 4). Daily GRO was also limited under wet soils and mild atmospheric demand (VPD = 0.5–1 kPa), attributable to sunny winter days when environmental conditions would be favourable for growth if phenological dormancy did not prevent it.

Species‐specific differences in daily GRO across the HMS were also observed. In the mesic site, *P. halepensis* showed the highest normalised GRO across most of the HMS, with a minimal hydrometeorological distance between spring and autumn conditions favourable for growth. *A. unedo* experienced the strongest growth limitations under comparable conditions, while the two oak species showed intermediate responses. Ecophysiological and phenological behaviours partly explain these differences among species. *P. halepensis* is a fast‐growing and early‐successional conifer species of low wood density (Table 2) adapted to Mediterranean climates (Camarero 2019). For *P. halepensis*, water availability in the mesic site seems optimum for allowing growth under broad hydrometeorological gradients, as similarly observed in a previous comparison among Mediterranean species (Sánchez‐Costa, Poyatos, and Sabaté 2015). The three angiosperm species showed substantial growth limitations at comparable conditions, suggesting that these prioritise carbohydrate storage over stem growth, as precisely observed for *Q. ilex* and *A. unedo* in inter‐specific meta‐analyses (Blumstein et al. 2022). Alternatively, they might allocate more carbon to root and leaf formation and maintenance metabolism. Among these angiosperm species, *A. unedo* showed the lowest GRO across most of the HMS, attributable to its high sensitivity to drought, observed at yearly time scales (Cherubini et al. 2003; Ogaya et al. 2003) and likely related to its limited ability to resume growth in autumn (Sánchez‐Costa, Poyatos, and Sabaté 2015). *Q. ilex* was capable of maintaining growth under soil drought, as reflected in its HMS by the fringe of dried soil (0.1 < REW < 0.25) above 0.5 kPa of VPD with relatively high GRO. This remarkable ability might be related to its late wood phenology, high potential for bimodal growth, high water use efficiency and deep root system (Campelo et al. 2018; Camarero, Rubio‐Cuadrado, and Gazol 2021). *Q. faginea* exhibited a similar behaviour to congeneric *Q. ilex*. However, its winter‐deciduous leaf habit might partly explain a comparatively lower tolerance to summer drought (Montserrat‐Martí et al. 2009).

In the xeric site, bimodal growth seasonality was not apparent across the HMS (Figure 5). *P. halepensis* responded bidirectionally to soil and atmospheric drought, while *J. thurifera* maintained low but stable GRO as long as the soil was minimally hydrated (REW > 0.1). Such contrasted behaviour might be partly explained from the iso/anisohydric perspective, commonly evaluated in ecophysiological studies with co‐existing pine and juniper species (e.g., McDowell et al. 2008). The relatively stable growth of (anisohydric) *J. thurifera* to VPD and REW compared to (isohydric) *P. halepensis* is consistent with observations on *Juniperus monosperma* and *Pinus edulis* where juniper trees maintained growth under broader drought stress gradients (Thompson et al. 2023). Likewise, when comparing *Larix decidua* and *Picea abies* in temperate climates, the growth of anisohydric larch trees was less sensitive to drought stress than spruce trees (Oberleitner et al. 2022). These observations suggest that the ability of anisohydric species to open stomata and maintain photosynthesis under soil and atmospheric drought facilitates the osmotic adjustment required to maintain cell turgor and growth under tolerable stress. As a trade‐off, anisohydric species may be exposed to more damaging dehydration levels under severe and prolonged drought, as suggested here by the larger TWD_MAX_ and TWD_CUM_ of *J. thurifera* (Figure 1g,h) despite its smaller diameter, likely attributable to the desiccation of stem living tissues.

### 4.3 Subdaily Variation in Growth and Dehydration

A dendrometer data set compiled over 8 years and 50 sites across Switzerland revealed that most stem growth GRO occurs during the night when VPD‐driven relaxation of stem water potential allows for the build‐up of cell turgor (Zweifel, Sterck, et al. 2021). The growth probability and contribution to annual GRO fluctuated from ca. 7% at nighttime to 2% at daytime, a pattern roughly consistent across the seven studied temperate species (Zweifel, Sterck, et al. 2021). Likewise, nighttime‐to‐daytime transitions of similar magnitude were consistently observed across 14 subtropical species (Zhou et al. 2023). Such contrasted nocturnal and diurnal growth patterns were inconsistent in the Mediterranean species studied here. Only mesic *P. halepensis* showed a threefold to fourfold reduction in growth probability and contribution to annual GRO during daytime (Figure 5, Table 3), similar to temperate and subtropical species. The remaining measurement sets experienced diurnal growth limitation; however, its magnitude was substantially lower, with nighttime‐to‐daytime ratios of GRO probability and contribution below 2.4, down to 1.2 in the case of *J. thurifera* in the xeric site.

The timing of stem shrinkage and swelling was assessed to explore the inter‐specific variability in sub‐daily growth ratios (Figure 6a). The first sub‐daily diameter maximum during growing days was not reached around dawn, except for mesic *P. halepensis*, the only case showing expected growth patterns driven by tree transpiration dynamics. Instead, local maxima were reached closer to solar midday for *Q. ilex*, *Q. faginea*, *A. unedo*, xeric *P. halepensis* and *J. thurifera*, with stems uniquely shrinking during the afternoon. Limited shrinkage upon resumption of transpiration denotes a remarkable ability of Mediterranean species to minimise and even avoid stem dehydration during the morning of growing days. Remarkably, the species‐specific ability to elude stem shrinkage during more hours was linearly related to their capacity to limit reductions in diurnal growth probability and contribution (Figure 6b,c). These relations suggest that the higher the stem resistance to shrink due to the capacitive water release to the transpiration stream (higher radial hydraulic resistance) during growing days, the higher the capacity to limit diurnal reductions of cell turgor and growth.

Consistent with nighttime‐to‐daytime growth ratios, the strength of circadian covariation of growth with VPD and REW decreased as follows: *P. halepensis* > *Q. ilex* > *Q. faginea* > *A. unedo* in the mesic site, and *P. halepensis* > *J. thurifera* in the xeric site (Figure 7). Overall, the circadian covariation was less apparent in the xeric site than in the mesic site, especially for *J. thurifera*. Moreover, when assessing hourly GRO across the HMS (Supporting Information: Figure S9), a predominant role of VPD over REW was observed in the mesic site, as similarly reported for temperate species (Zweifel, Sterck, et al. 2021), and attributed to the overnight xylem tension relaxation and subsequent build‐up of cell turgor. The predominance of VPD over REW was not that apparent in the xeric site, where hourly GRO responded bidirectionally to both VPD and REW. Attenuated circadian patterns and increasing relevance of REW in the xeric site suggest that, as site aridity increases, overnight refilling, cell turgor build‐up and growth are substantially constrained during most of the growing season, thereby attenuating nighttime‐to‐daytime growth transitions. Contrastingly, seasonal soil water depletion might be incomplete in wetter sites during most of the growing season, facilitating overnight stem refilling and growth, thereby intensifying circadian growth patterns. Remarkably, hourly GRO (Supporting Information: Figure S9) and daily GRO (Figure 4) across the HMS substantially differed, highlighting the critical relevance of the temporal scale considered for analyses. At the hourly time scale, GRO reflects the nearly instantaneous conditions of stem water status and cell turgor, determining the sink growth demand. At the daily time scale, GRO depends on both nighttime and daytime conditions, including diurnal photosynthetic hours, thus implicitly integrating information about the sink and source balance.

### 4.4 How Might Mediterranean Species Maintain Diurnal Growth?

The prevailing hypothesis that stem growth is restricted to the nighttime derives mainly from observations in temperate species. Mediterranean trees monitored here also grew more during the night (ratios > 1); however, our observations challenge the expectation of a major impediment to growth during the daytime. At least, they challenge consistent patterns across species (cf. Zhou et al. 2023): mesic *P. halepensis* showed the expected diurnal growth reductions, while *J. thurifera*, *Q. faginea* and *A. unedo* clearly maintained non‐negligible diurnal growth (ratios < 1.6). Although scarce, reports of significant diurnal growth can be found in the literature. For Mediterranean species, *J. oxycedrus* seedlings subjected to drought stress swelled during the daytime (Mayoral et al. 2016), the alpine shrub *Genista versicolor* only swelled after midday during wet growing conditions (Albrecht et al. 2023), and the daytime growth reduction of *J. excelsa* was limited compared to *Cedrus libani* montane trees (Güney et al. 2020). In the mangrove species *Avicennia marina*, daytime stem swelling, occurring year‐round, was related to light availability for photosynthesis (Donnellan Barraclough et al. 2018). Further evidence challenges the assumption of predominant nighttime stem vertical growth observed across species and seasons, with growth peaks often occurring in the afternoon (see Table 2 in Mencuccini et al. 2017). Although not measured here, all these observations point to the relevant role of carbon gain and osmoregulation in buffering diurnal growth reductions in seasonally dry environments.

Species from Mediterranean climates commonly experience lower seasonality in nonstructural carbohydrate concentrations than temperate ones (Martínez‐Vilalta et al. 2016). Soluble sugars and phloem osmolality in stems and branches have been observed to be lowest in mid‐latitude (temperate) regions and increase toward more arid (and colder) regions (Lintunen et al. 2016; Blumstein et al. 2023). These studies suggest the critical role of osmolytes in tolerating environmental stress (see also Long and Adams 2022), which might be particularly relevant in Mediterranean environments. A high osmolyte concentration in stem living cells facilitates the maintenance of cell turgor to limit lethal dehydration during summer (Rodríguez‐Calcerrada et al. 2020; Blumstein et al. 2023), potentially conferring the ability to buffer diurnal growth reductions under more favourable (wetter) conditions. Model simulations across several tree species reflected a variable fraction of stem growth occurring during daytime depending on case‐specific source‐sink scenarios (Mencuccini et al. 2017; Pfautsch et al. 2021; Salomón, De Roo et al. 2022), providing mechanistic support to osmotically‐driven swelling when phloem loading exceeds the sink demand for growth. To empirically demonstrate the osmoregulatory role in sub‐daily stem growth trends, a better characterisation of sub‐daily osmolyte dynamics would be required. Such studies, however, remain scarce due to the technical difficulty of monitoring osmolytes and carbohydrate concentrations at a sub‐hourly temporal resolution comparable to that of automatic dendrometers. The few studies available point to a stronger osmotic signal in bark thickness as we move from the tree trunk (sink) towards the canopy (source) (Lazzarin et al. 2019; Epron et al. 2021). For future studies, we encourage the compilation of highly‐resolved dendrometer data sets across broad gradients of site aridity to test potential relations of sub‐daily growth ratios with osmoregulatory and hydraulic traits.

## 5 Conclusions

Stem diameter variations of Mediterranean species partially deviated from our current understanding of tree stem growth and dehydration patterns, mainly derived from observations in temperate species. Annually, growth was consistently explained across species by the time windows favourable for growth, primarily restricted by summer soil drought within the growing season. Seasonally, the strength of the bimodal growth patterns varied with the species' ecology and site hydrometeorological conditions. Sub‐daily, a substantial fraction of stem growth occurred during the daytime when soil drought did not exceed the threshold of seasonal shrinkage. The maintenance of diurnal growth suggests a relevant role of osmoregulation in avoiding dehydration and maintaining cell turgor during transpiration hours. Overall, our observations anticipate that more intense and longer summer drought events expected in Mediterranean regions will substantially limit tree stem growth. These limitations will be determined by the onset of the soil summer drought, shortening the spring growth window and the drought extension into autumn, limiting the capability of Meditteranean species to resume growth.

## Conflicts of Interest

The authors declare no conflicts of interest.

## Supporting information

Supporting information.

## Data Availability

The data that support the findings of this study are available from the corresponding author upon reasonable request.
