# Supplementary material for: Stem Growth and Dehydration Responses of Mediterranean Tree Species to Atmospheric and Soil Drought
Source: Plant Cell Environ. 2024 Oct 3;48(1):866–81. doi: 10.1111/pce.15177 (PMC11615415; doi:10.1111/pce.15177)
Supplement: Supplementary file 1 — Supporting information. [file PCE-48-866-s001.pdf]

Supporting information

Stem growth and dehydration responses of Mediterranean tree species to atmospheric and soil drought

Roberto L. Salomón[1], J. Julio Camarero[2]

[1] Departamento de Sistemas y Recursos Naturales, Research Group FORESCENT, Universidad Politécnica de Madrid, Jose Antonio Novais 10, 28040, Madrid, Spain

[2] Instituto Pirenaico de Ecología (IPE-CSIC), Avda. Montañana 1005, 50059 Zaragoza, Spain

Fig. S1. Location of the study sites in the Iberian Peninsula (a) and aerial views and climate diagrams (1993-2022 period) of (b) Agüero and (c) Peñaflor study sites. In plot (a), the green background shows the forest cover.

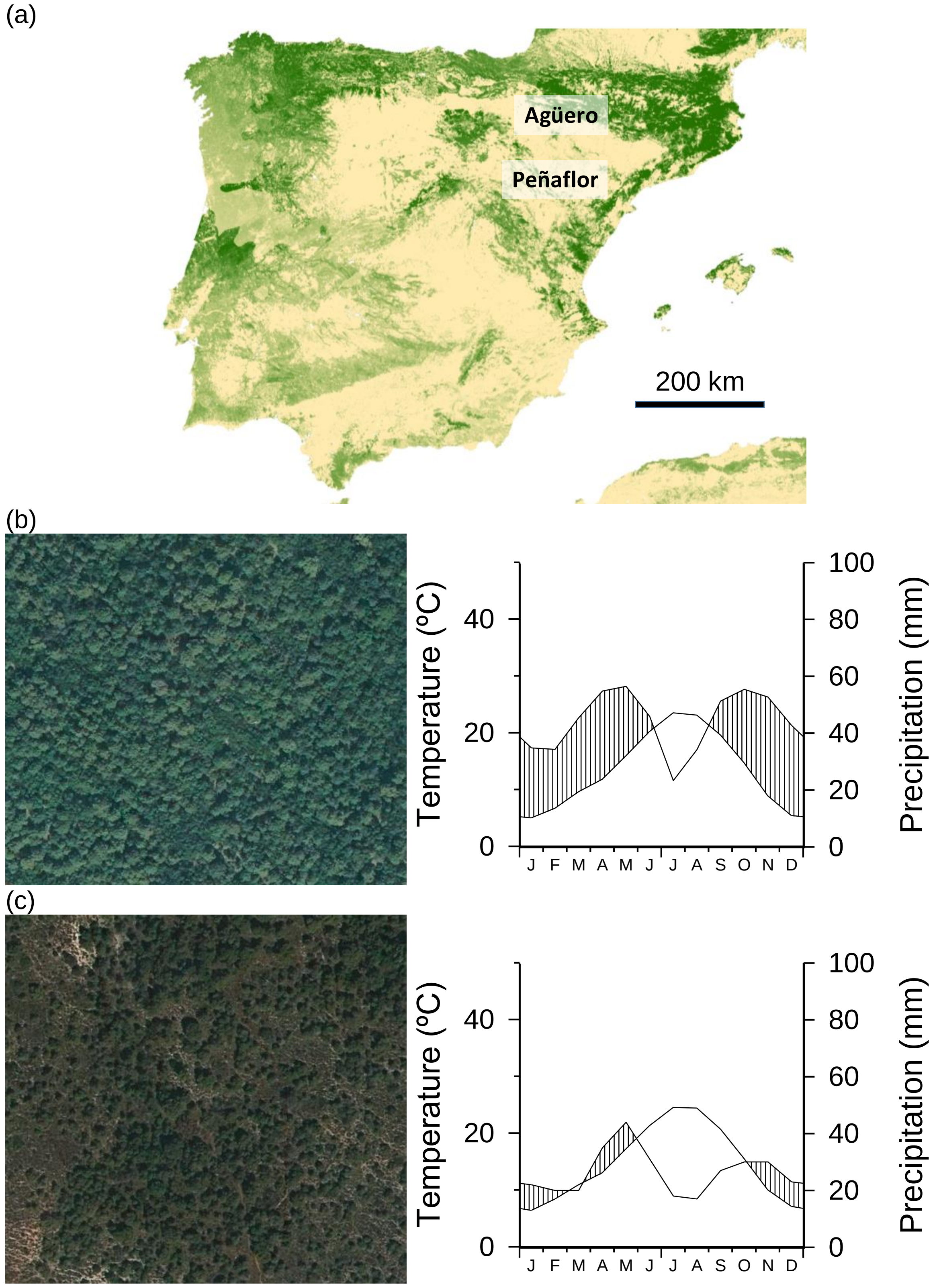

Fig. S2. Climatic data in the mesic site (Agüero).

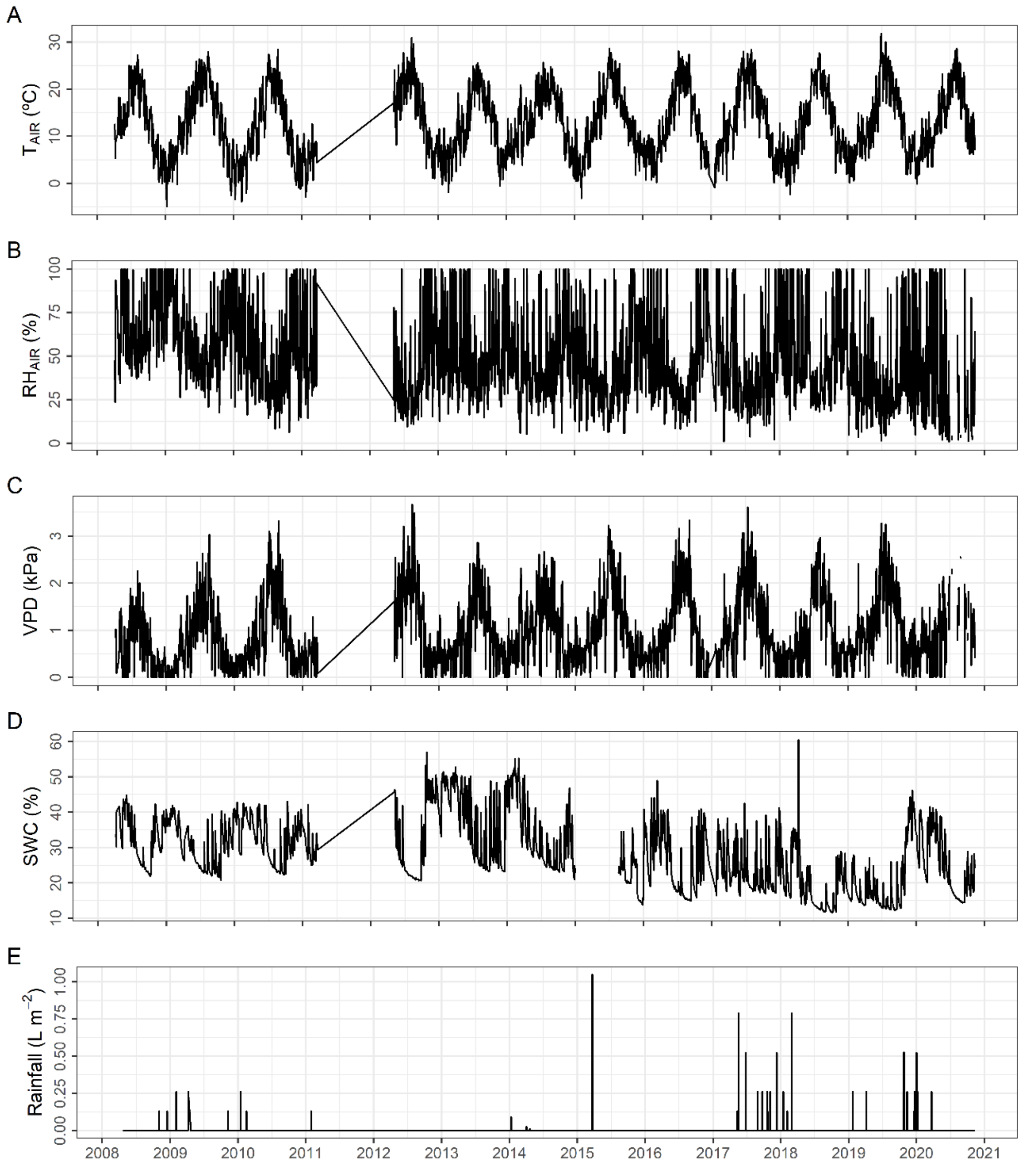

Climatic data includes air temperature (T_{AIR}, A), air relative humidity (RH_{AIR}, A), vapour pressure deficit(VPD, C), soil water content (SWC, D), and rainfall (E).

Fig. S3. Climatic data in the xeric site (Peñaflor).

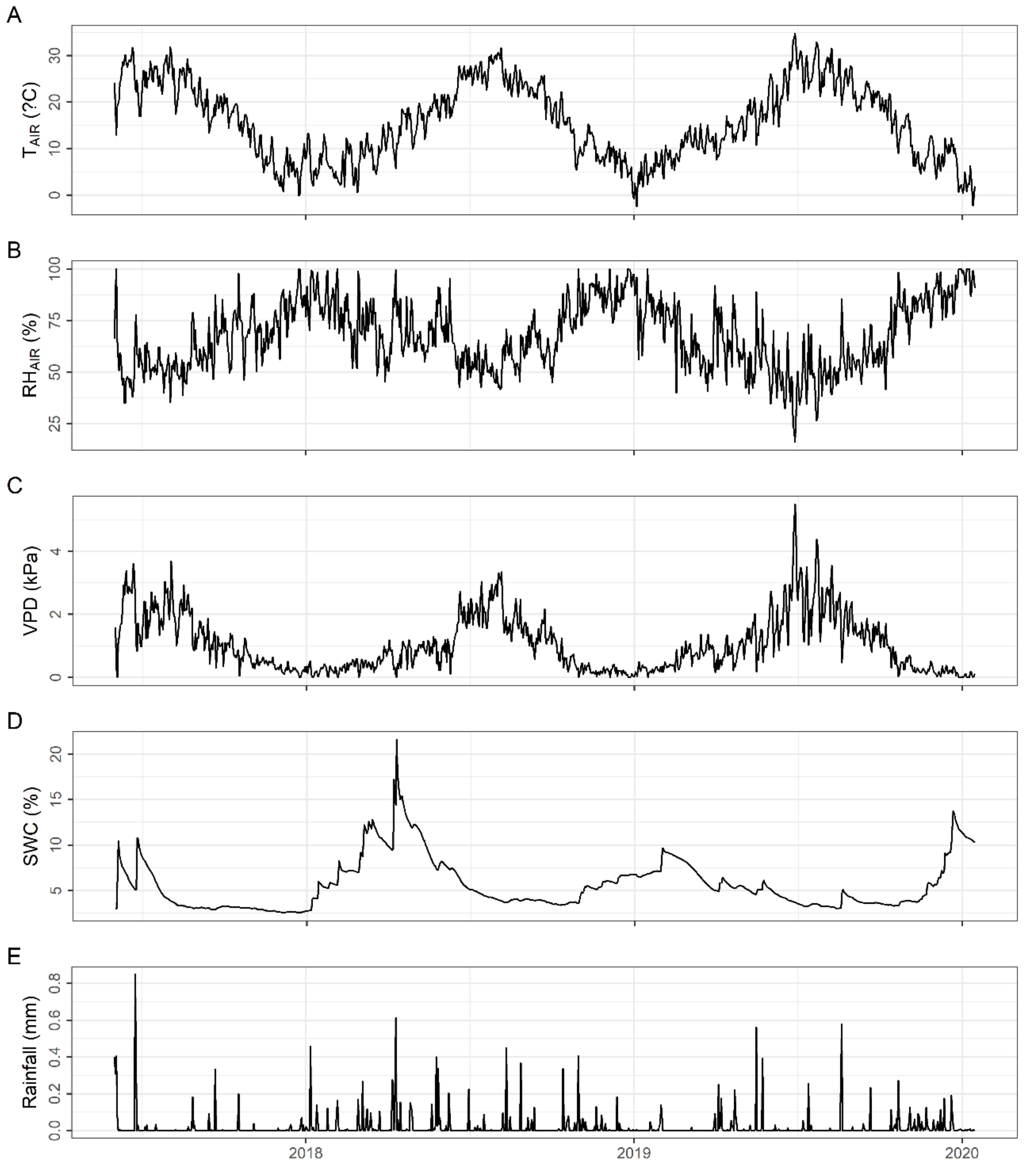

Climatic data includes air temperature (T_{AIR}, A), air relative humidity (RH_{AIR}, A), vapour pressure deficit(VPD, C), soil water content (SWC, D), and rainfall (E).

Fig. S4. Dendrometer data output (growth and tree water deficit, TWD) from the mesic site (period 2009-2014) after cleaning and processing with the "*treenetproc*" package.

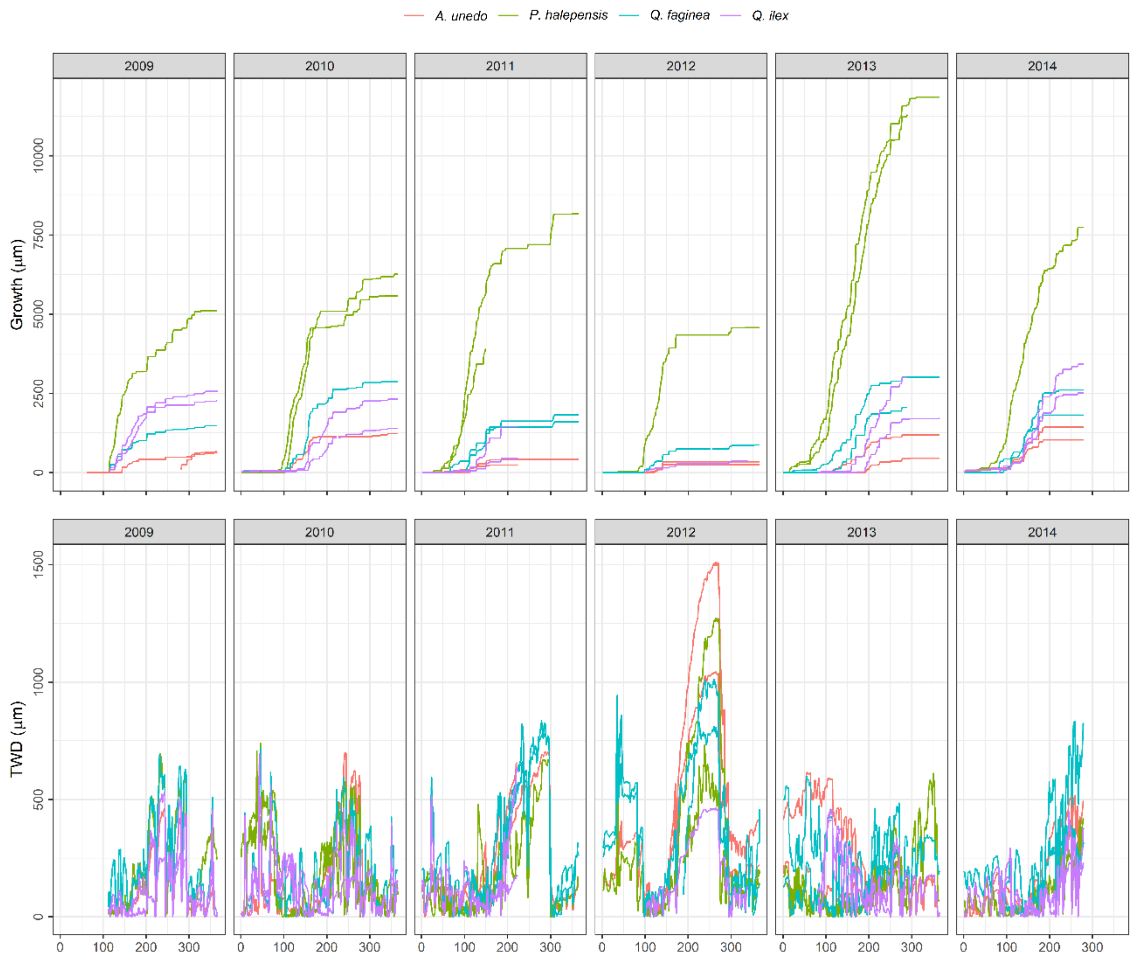

Two individuals per species were monitored in the mesic site for six years. The horizontal axes show the day of the year (DOY).

Fig. S5. Dendrometer data output (growth and tree water deficit, TWD) from the xeric site (periods 2009-2013 and 2016-22) after cleaning and processing with the "*treenetproc*" package.

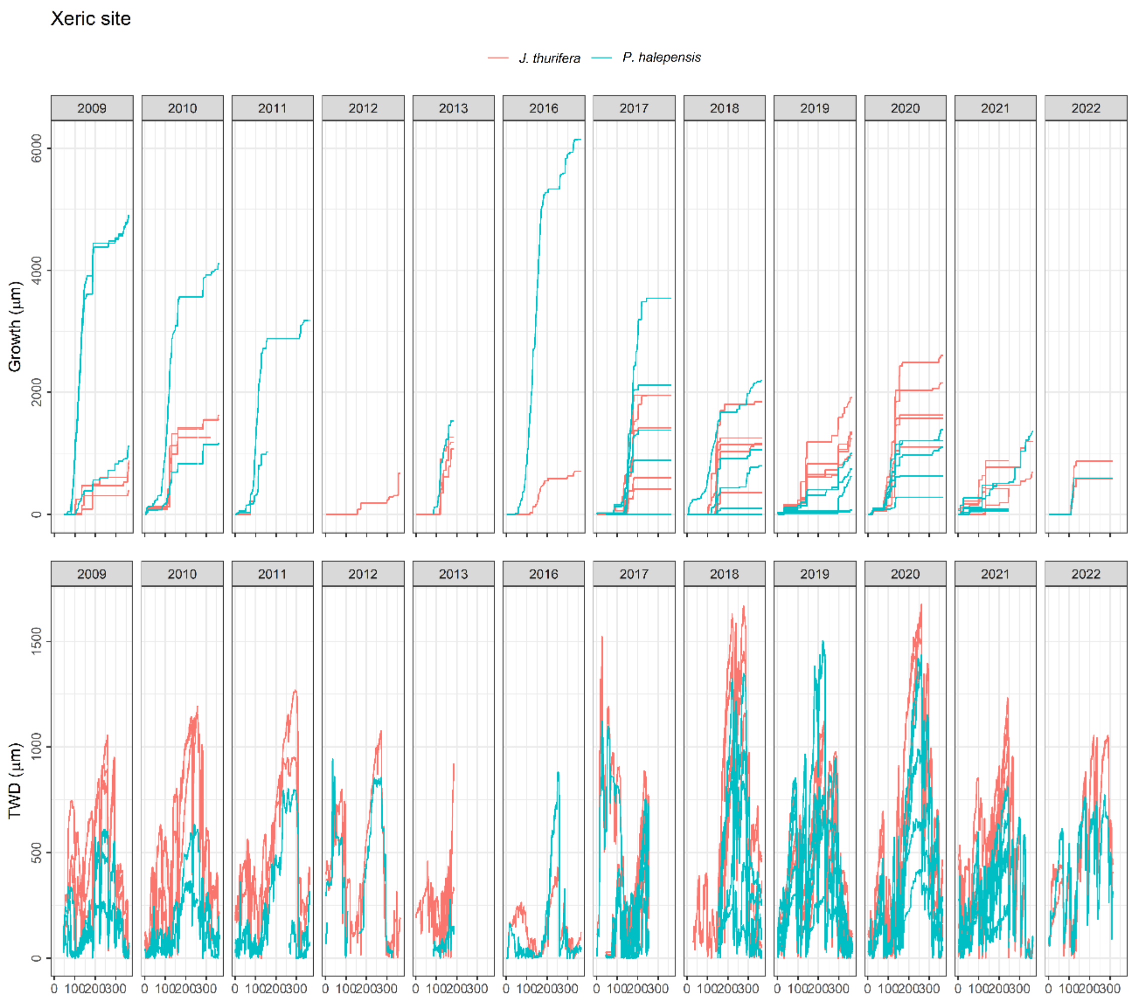

Five or six individuals per species were monitored in the xeric site for 12 years. The horizontal axes show the day of the year (DOY).

Fig. S6. Amount of variance explained by relative extractable water (REW), vapour pressure deficit (VPD), their interaction (REW:VPD), and their additive effect (REW+VPD) on normalized daily growth (GRO) and tree water deficit (TWD) on a daily basis.

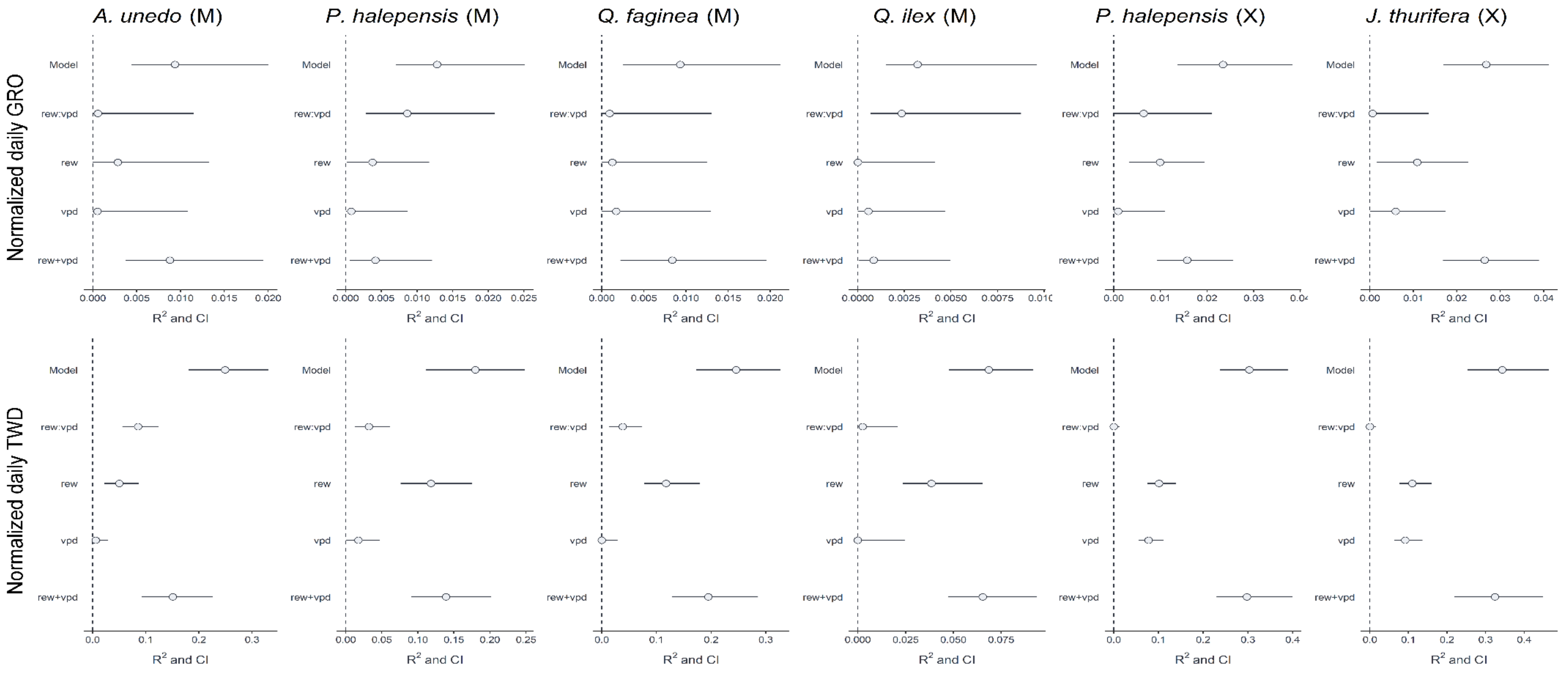

R^2 values and the confidence intervals were obtained with the "partR2" package. Note that we report the (non-inclusive) proportion of variance explained by a main effect, together with the variance jointly explained by its interaction with the main effect (the so-called "Option C" in Stoffel et al. 2021).

Fig. S7. Hydrometeorological space (HMS), defined by vapour pressure deficit (VPD) and relative extractable water (REW), considering median daily values per species and site.

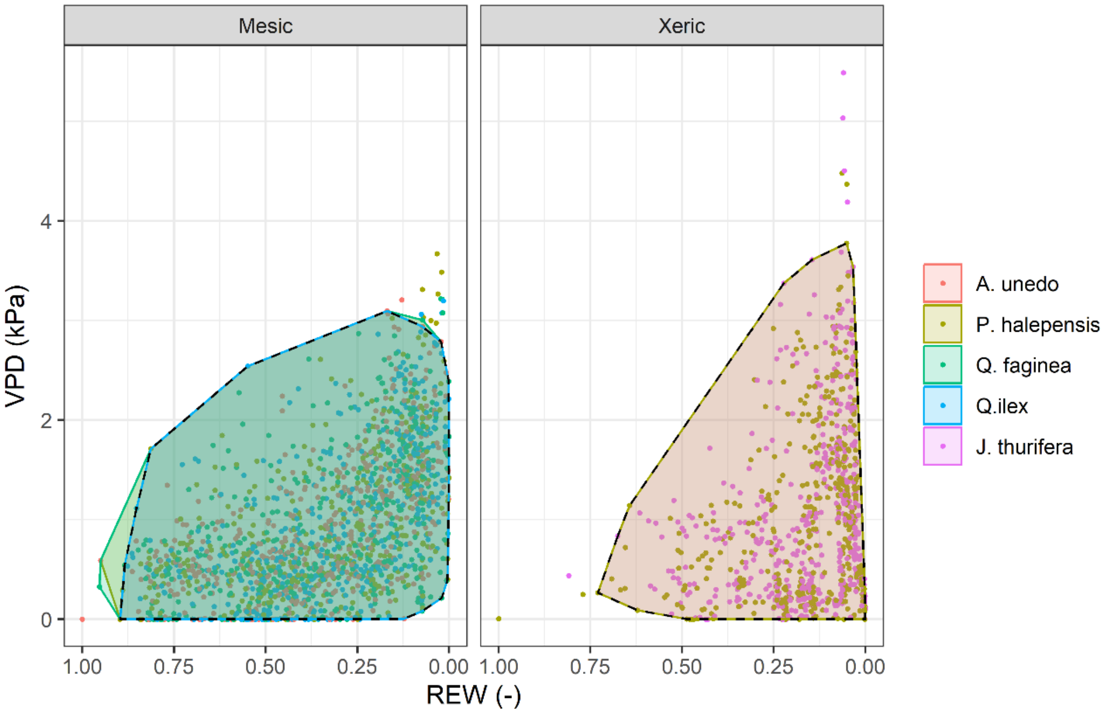

The dashed polygon depicts the common HMS shared by all species per site, which is considered for modelling purposes. The furthest (99th percentile) conditions from the HMS centre were considered outliers (outside the polygons) and omitted from the modelling.

Fig. S8. Sub-daily patterns of stem diameter variations (ΔD) zeroed at midnight considering all days during the study period.

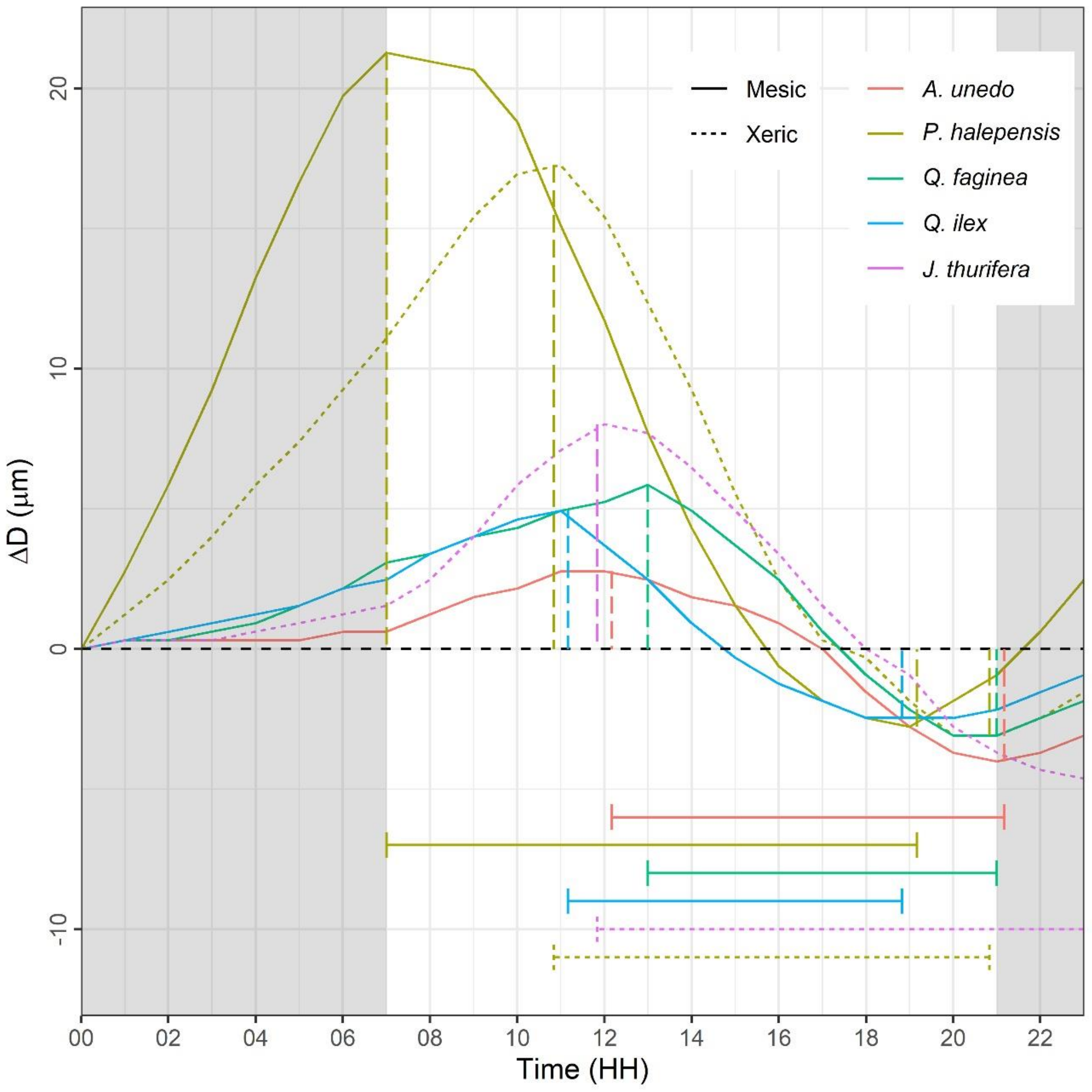

Subdaily extremes (vertical dashed lines) delimiting the shrinkage period (horizontal segments) are shown.

Fig. S9. Normalized hourly growth across the hydrometeorological space, defined by vapour pressure deficit (VPD) and relative extractable water (REW).

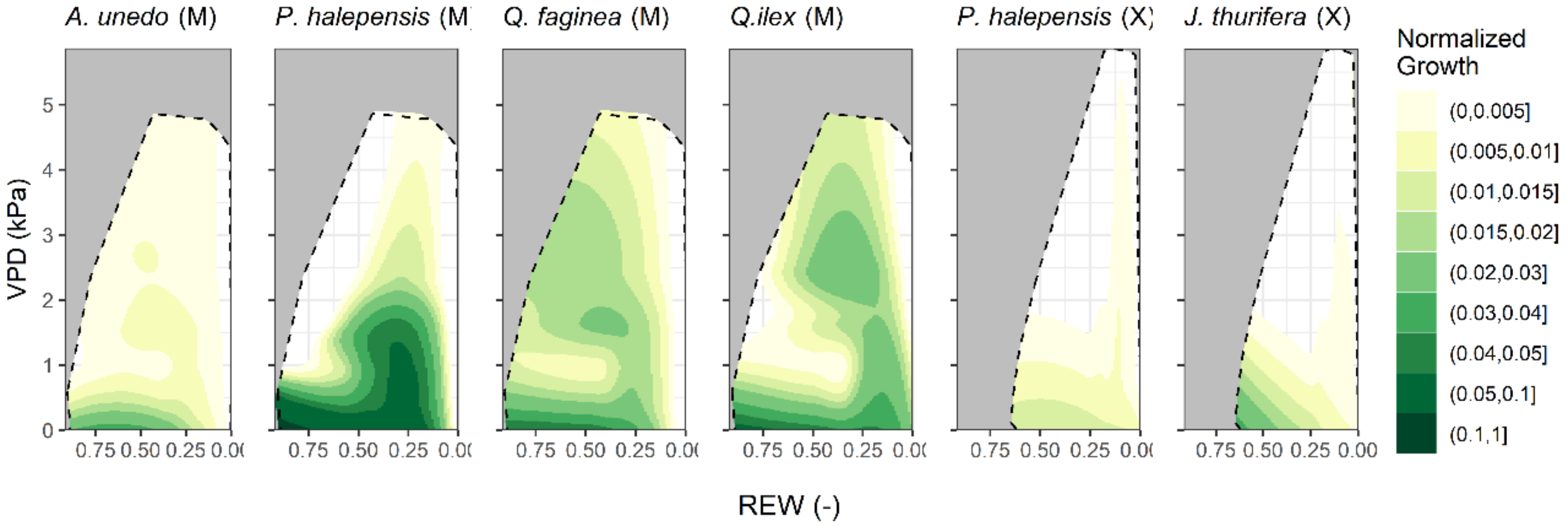

Normalized growth is relative to the maximum (99th percentile) per tree during the study period.

Notes S1. Code of the mixed models used for analyses.

Yearly analyses:

lmer(annual_GRO ~ x_i + (1|tree), data=db_i)

lmer(annual_GRO ~ x_i + (1|tree), data=db)

being x_i GRO_{START}, GRO_{END}, GRO_{LENGTH}, GRO_{DAYS}, GRO_{HOURS}, TWD_{MAX}, TWD_{CUM}, TWD:GRO_{LENGTH}, REW_{CUM} and VPD_{CUM}, and db and db_i the whole dataset or each species:site dataset, respectively.

When annual GRO was regressed against climatic drivers:

lmer(annual_GRO ~ vpd_cum + (1|tree), data=db)

lmer(annual_GRO ~ rew_cum + (1|tree), data=db)

Seasonal analyses:

For marginal R^2 partitioning of climatic drivers (see so-called option C in Stoffel et al. 2021).

*m1<- lmer(normalized daily GRO ~ rew * vpd + (1|year) + (1|tree), data=db_i)*

p1 <- partR2(m1, partvars = c("rew:vpd"), nboot=100)

m2<-lmer(normalized daily GRO ~ rew + vpd + (1|year) + (1|tree), data= db_i)

p2 <- partR2(m2, partvars = c("rew","vpd"), nboot=100)

mergeR2(p1, p2)

Identical analyses were made for the *normalized daily TWD* as the dependent variable.

For modelling GRO and TWD across the hydrometeorological space:

*loess(normalized daily GRO ~ rew * vpd, data = db_i, span= 0.75, degree = 2)*

*loess(normalized daily TWD ~ rew * vpd, data = db_i, span= 0.75, degree = 2)*

Subdaily analyses:

*loess(normalized hourly GRO ~ hour * vpd, data = db_i, span= 0.75, degree = 2)*

*loess(normalized hourly GRO ~ hour * rew, data = db_i, span= 0.75, degree = 2)*

*loess(normalized hourly GRO ~ vpd * rew, data = db_i, span= 0.75, degree = 2)*
